# Immune-tumor interaction dictates spatially directed evolution of esophageal squamous cell carcinoma

**DOI:** 10.1093/nsr/nwae150

**Published:** 2024-04-23

**Authors:** Yong Zhou, Shanlan Mo, Heyang Cui, Ruifang Sun, Weimin Zhang, Xiaofei Zhuang, Enwei Xu, Hongyi Li, Yikun Cheng, Yongsheng Meng, Meilin Liu, Ting Yan, Huijuan Liu, Ling Zhang, Bin Yang, Yanfeng Xi, Shubin Wang, Xiaolong Cheng, ShuaiCheng Li, Zhihua Liu, Qimin Zhan, Zheng Hu, Yongping Cui

**Affiliations:** Cancer Institute, Department of Pathology, Peking University Shenzhen Hospital, Shenzhen Peking University-the Hong Kong University of Science and Technology (PKU-HKUST) Medical Center; Institute of Cancer Research, Shenzhen Bay Laboratory, Shenzhen 518000, China; Key Laboratory of Cellular Physiology of the Ministry of Education, Department of Pathology, Shanxi Medical University, Taiyuan 030001, China; City University of Hong Kong Shenzhen Research Institute, Shenzhen 518000, China; CAS Key Laboratory of Quantitative Engineering Biology, Shenzhen Institute of Synthetic Biology, Shenzhen Institute of Advanced Technology, Chinese Academy of Sciences, Shenzhen 518000, China; Cancer Institute, Department of Pathology, Peking University Shenzhen Hospital, Shenzhen Peking University-the Hong Kong University of Science and Technology (PKU-HKUST) Medical Center; Institute of Cancer Research, Shenzhen Bay Laboratory, Shenzhen 518000, China; Key Laboratory of Cellular Physiology of the Ministry of Education, Department of Pathology, Shanxi Medical University, Taiyuan 030001, China; Department of Surgery, Faculty of Medicine, The Chinese University of Hong Kong, Hong Kong 999077, China; Department of Tumor Biobank, Shanxi Province Cancer Hospital/Shanxi Hospital Affiliated to Cancer Hospital, Chinese Academy of Medical Sciences/Cancer Hospital Affiliated to Shanxi Medical University, Taiyuan 030013, China; Cancer Institute, Department of Pathology, Peking University Shenzhen Hospital, Shenzhen Peking University-the Hong Kong University of Science and Technology (PKU-HKUST) Medical Center; Institute of Cancer Research, Shenzhen Bay Laboratory, Shenzhen 518000, China; Key Laboratory of Carcinogenesis and Translational Research (Ministry of Education/Beijing), Laboratory of Molecular Oncology, Peking University Cancer Hospital & Institute, Beijing 100142, China; Research Unit of Molecular Cancer Research, Chinese Academy of Medical Sciences, Beijing, China; Department of Thoracic Surgery, Shanxi Province Cancer Hospital/Shanxi Hospital Affiliated to Cancer Hospital, Chinese Academy of Medical Sciences/Cancer Hospital Affiliated to Shanxi Medical University, Taiyuan 030013, China; Department of Pathology, Shanxi Province Cancer Hospital/Shanxi Hospital Affiliated to Cancer Hospital, Chinese Academy of Medical Sciences/Cancer Hospital Affiliated to Shanxi Medical University, Taiyuan 030013, China; Key Laboratory of Cellular Physiology of the Ministry of Education, Department of Pathology, Shanxi Medical University, Taiyuan 030001, China; Cancer Institute, Department of Pathology, Peking University Shenzhen Hospital, Shenzhen Peking University-the Hong Kong University of Science and Technology (PKU-HKUST) Medical Center; Institute of Cancer Research, Shenzhen Bay Laboratory, Shenzhen 518000, China; College of Letters & Science, University of California Berkeley, Berkeley, CA 94704, USA; Department of Tumor Biobank, Shanxi Province Cancer Hospital/Shanxi Hospital Affiliated to Cancer Hospital, Chinese Academy of Medical Sciences/Cancer Hospital Affiliated to Shanxi Medical University, Taiyuan 030013, China; Department of Tumor Biobank, Shanxi Province Cancer Hospital/Shanxi Hospital Affiliated to Cancer Hospital, Chinese Academy of Medical Sciences/Cancer Hospital Affiliated to Shanxi Medical University, Taiyuan 030013, China; Key Laboratory of Cellular Physiology of the Ministry of Education, Department of Pathology, Shanxi Medical University, Taiyuan 030001, China; Key Laboratory of Cellular Physiology of the Ministry of Education, Department of Pathology, Shanxi Medical University, Taiyuan 030001, China; Key Laboratory of Cellular Physiology of the Ministry of Education, Department of Pathology, Shanxi Medical University, Taiyuan 030001, China; Department of Thoracic Surgery, Shanxi Province Cancer Hospital/Shanxi Hospital Affiliated to Cancer Hospital, Chinese Academy of Medical Sciences/Cancer Hospital Affiliated to Shanxi Medical University, Taiyuan 030013, China; Department of Pathology, Shanxi Province Cancer Hospital/Shanxi Hospital Affiliated to Cancer Hospital, Chinese Academy of Medical Sciences/Cancer Hospital Affiliated to Shanxi Medical University, Taiyuan 030013, China; Cancer Institute, Department of Pathology, Peking University Shenzhen Hospital, Shenzhen Peking University-the Hong Kong University of Science and Technology (PKU-HKUST) Medical Center; Institute of Cancer Research, Shenzhen Bay Laboratory, Shenzhen 518000, China; Key Laboratory of Cellular Physiology of the Ministry of Education, Department of Pathology, Shanxi Medical University, Taiyuan 030001, China; City University of Hong Kong Shenzhen Research Institute, Shenzhen 518000, China; State Key Laboratory of Molecular Oncology, National Cancer Center/National Clinical Research Center for Cancer/Cancer Hospital, Chinese Academy of Medical Sciences and Peking Union Medical College, Beijing 100021, China; Cancer Institute, Department of Pathology, Peking University Shenzhen Hospital, Shenzhen Peking University-the Hong Kong University of Science and Technology (PKU-HKUST) Medical Center; Institute of Cancer Research, Shenzhen Bay Laboratory, Shenzhen 518000, China; Key Laboratory of Carcinogenesis and Translational Research (Ministry of Education/Beijing), Laboratory of Molecular Oncology, Peking University Cancer Hospital & Institute, Beijing 100142, China; Research Unit of Molecular Cancer Research, Chinese Academy of Medical Sciences, Beijing, China; CAS Key Laboratory of Quantitative Engineering Biology, Shenzhen Institute of Synthetic Biology, Shenzhen Institute of Advanced Technology, Chinese Academy of Sciences, Shenzhen 518000, China; Cancer Institute, Department of Pathology, Peking University Shenzhen Hospital, Shenzhen Peking University-the Hong Kong University of Science and Technology (PKU-HKUST) Medical Center; Institute of Cancer Research, Shenzhen Bay Laboratory, Shenzhen 518000, China; Key Laboratory of Cellular Physiology of the Ministry of Education, Department of Pathology, Shanxi Medical University, Taiyuan 030001, China

**Keywords:** squamous cell carcinoma, heterogeneity, multi-omics, evolution, tumor microenvironment

## Abstract

Esophageal squamous cell carcinoma (ESCC) is a poor-prognostic cancer type with extensive intra- and inter-patient heterogeneity in both genomic variations and tumor microenvironment (TME). However, the patterns and drivers of spatial genomic and microenvironmental heterogeneity of ESCC remain largely unknown. Here, we generated a spatial multi-omic atlas by whole-exome, transcriptome, and methylome sequencing of 507 tumor samples from 103 patients. We identified a novel tumor suppressor *PREX2*, accounting for 22% of ESCCs with frequent somatic mutations or hyper-methylation, which promoted migration and invasion of ESCC cells *in vitro*. Analysis of the TME and quantification of subclonal expansion indicated that ESCCs undergo spatially directed evolution, where subclones mostly originated from the tumor center but had a biased clonal expansion to the upper direction of the esophagus. Interestingly, we found upper regions of ESCCs often underwent stronger immunoediting with increased selective fitness, suggesting more stringent immune selection. In addition, distinct TMEs were associated with variable genomic and clinical outcomes. Among them, hot TME was associated with high immune evasion and subclonal heterogeneity. We also found that immunoediting, instead of CD8^+^ T cell abundance, acts as an independent prognostic factor of ESCCs. Importantly, we found significant heterogeneity in previously considered potential therapeutic targets, as well as BRCAness characteristics in a subset of patients, emphasizing the importance of focusing on heterogeneity in ESCC targeted therapy. Collectively, these findings provide novel insights into the mechanisms of the spatial evolution of ESCC and inform precision therapeutic strategies.

## INTRODUCTION

Single- and multiple-sample analyses have shown that tumor displays both extensive intra- and inter-patient heterogeneity [1]. The heterogeneity is reflected by the diversity of the local genetic and tumor microenvironment. Genetic and tumor microenvironment (TME) heterogeneity is associated with therapeutic resistance and tumor relapse [2]. Multi-regional sequencing (MRS) has been a useful approach to characterize tumor heterogeneity and depict spatiotemporal evolution. Understanding the genomic alterations and TME by aggregating multi-regional data might facilitate the identification of evolutionary patterns thus leading to more effective therapeutics.

However, most of the large-scale studies on esophageal squamous cell carcinoma (ESCC) are based on single-site sampling [3–6], which might underestimate the heterogeneity and is inefficient to probe the mechanisms of spatial heterogeneity. Given that tumor evolves through both space and time, many patients have demonstrated substantial heterogeneity accompanied with lymph-node metastasis. Moreover, in addition to the oncogenic mutations, epigenetic change can also be the driver for tumor initiation and progression [7]. For instance, hypermethylation of tumor suppressors was frequently present in ESCCs [8]. Nevertheless, integrating both genetic and epigenetic changes in ESCC was less studied than that in other human cancers. It is necessary to chart a landscape at the patient level to refine the genetic and epigenetic basis of ESCC.

Further, a few studies have described the genetic heterogeneity within ESCC through multi-regional sequencing of pre-cancerous or advanced tumor samples [9–11]. These studies unveiled substantial heterogeneity of genomic alterations in ESCCs, especially for actionable targets such as *EGFR, PD-L1*, and *CDK6*. They consistently revealed that branched evolution is the predominated pattern for clonal diversification in ESCCs. However, these studies were limited to a small number of patients, often consisting of dozens of patients. The impact of tumor geography on subclonal evolution is not systematically studied in ESCC either. Specifically, tumor mass locates vertically at the surface of the esophagus. Thus, samples of different regions, for instance, samples from the bottom and the upper of the tumor mass, might suffer from differential pressure. The cause for differential pressure might come from the different extent of stimulation from digestive food to the bottom and upper regions of the tumor mass when food flows through the tumor mass. Distinct pressures might lead to spatial differences for subclonal migration and expansion. Although the heterogeneity between the tumor center and the marginal region was widely noticed in human cancer [12], the spatial heterogeneity of distinct specific regions within ESCC was not yet clear. In addition, how subclones geographically grow to different regions of tumor mass during branched evolution is unknown.

To spatially explore the heterogeneity of ESCCs, we employed a multi-regional sampling and multi-omics (whole-exome sequencing or WES, RNA-seq, and methylation array) strategy for the primary tumor and matched lymph node (LN) metastasis. Especially, the samples from each tumor have well-annotated spatial information in terms of the directions in the esophagus (bottom/upper/left/right of the tumor mass). We aimed to generate a genetic map of ESCC at the patient level and explore the genetic, epigenetic, and microenvironment heterogeneity within ESCC. We found distinct mutational scenarios compared to single-site sequencing, characterized by high-frequency driver mutations in the ESCC population and a novel driver, *PREX2*, further verified by *in vitro* experiments. In addition, we proposed a spatially directed model for ESCC evolution, where subclones of the tumor center tended to grow towards a specific direction of the upper esophagus. We also found that immunoediting ability largely depended on the context of immune cell infiltrates and could act as an independent prognostic factor. BRCAness ESCCs demonstrate better outcomes in three independent data cohorts, indicating that these subset patients might benefit from platinum-based chemotherapy and PARP inhibitors.

## RESULTS

### Sample characterization

**Figure 1.**
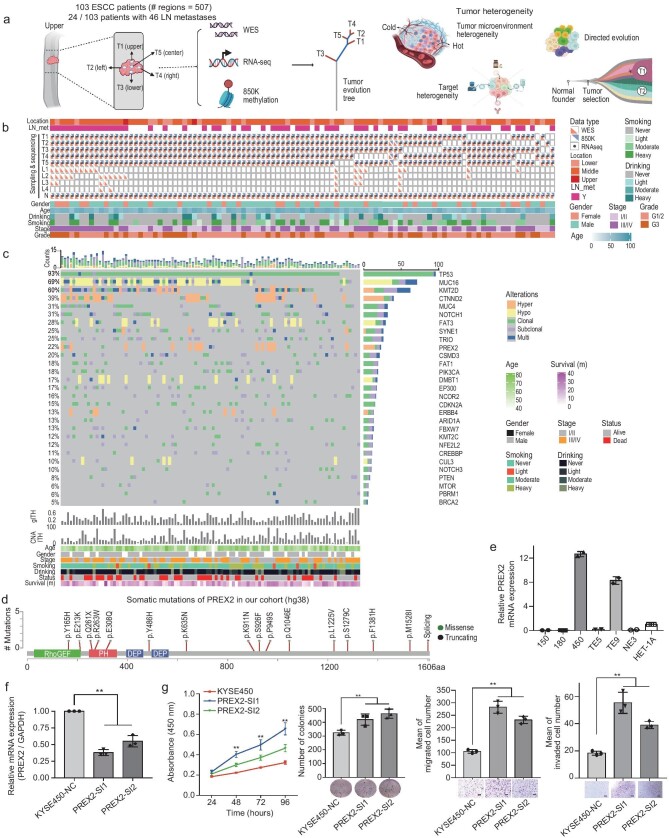
Integrated mutational landscape of 103 ESCC patients in this study. (a) The sampling strategy of regional tumor and lymph-node metastasis. The upper, bottom, left, and right side of the primary tumor were shown. (b) Per-patient overview of sampling and clinical information across 103 ESCCs. The location and lymphatic metastasis are annotated at the top and other clinical characters (gender, age, drinking, smoking, stage, and grade) at the bottom. The middle described the sequenced regions for primary tumors as panel A and the numbers of lymph-node metastases in detail. (c) The landscape of frequently altered genes driven by genetic and epigenetic changes for the ESCC cohort. (d) Schematic diagram of *PREX2* mutations. (e) Bar-plot of *PREX2* expression in different ESCC cell lines. (f) Bar-plot of knockdown efficiency of *PREX2*. (g) Cell function experiments of *PREX2* in ESCC cell line KYSE450. Left: The line plot displays the proliferation of cells after *PREX2* knock-down by MTT assay and the bar plot displays the number of colonies in cells. Right: The Transwell cell migration and invasion assay after *PREX2* knock-down. Scale bars, 200 μm. All bar-plots are presented as the mean ± standard deviation. Three independent experiments were performed; each experiment was performed in triplicate. Statistical analysis is performed with one-way ANOVA. *P ≤ 0.05, **P ≤ 0.01.

We collected 507 tumor samples, consisting of 461 primary tumor regions and 46 LN metastases from 103 patients with matched adjacent normal samples through MRS for whole exome, transcriptome, and methylome simultaneously (Fig. 1a, Supplementary Data 1, 2). None of the 103 patients underwent adjuvant therapy before surgery. In particular, 2 to 5 regions were sequenced for each of the primary tumors. Forty-two ESCC patients were diagnosed as early-stage (stage I & II), while 61 patients were diagnosed as advanced-stage (stage III & IV). The average diagnosis age was 63 years-old, ranging from age 40 to 80 years-old (Supplementary Data 1). In our dataset, the regional samples were collected from five locations of the primary tumor: center (T5), upper (to the oral cavity, T1), lower (to the stomach, T3), and left/right (T2/T4). Due to the tumor's three-dimensional properties, we realized that it is meaningless to distinguish between the left and right sides (Supplementary Data 2). Nevertheless, the tumor center, upper and lower sides were in real anatomical directions (Fig. 1a). LN metastases were sampled when available. Tumors and adjacent normal tissues were subjected to WES, methylation, and RNA-seq (Fig. 1b). We achieved a high average sequencing depth for the WES of primary tumors (*n* = 461; median depth: 310.3X; 95% CI: 234.4–379.5) and LN metastases (*n* = 46; median depth: 340X; 95% CI: 224.3–450.9) (Fig. S1a and Supplementary Data 2). The workflow of this study is shown in Fig. S1b.

### The integrated mutational landscape and heterogeneity across 103 ESCCs

Mutect2 [13] was applied to detect somatic mutations in this ESCC cohort (Supplementary Notes). As a result, we detected a total of 35 972 unique non-synonymous mutations at the patient level (Supplementary Data 3a), with a median of 8.2 unique somatic mutations per megabase (Mb; range: 2.9–141 mutations/Mb) for each patient. The mutation burden at the patient level (unique mutations across all regions) was significantly higher than that in a single sample (*P* < 0.001, Fig. S2a). Previously, patients with mutation burden (≥10 mutations/Mb) were defined as having a high mutation burden [14]. This criterion might need to be revised at the patient level, as 35% of ESCC patients met this criterion in our cohort. Three hyper-mutated patients with 141, 92, and 81 mutations/Mb, respectively, ESCC012, ESCC049, and ESCC061 (Supplementary Data 3b), were identified in our cohort.

The mutational processes within 103 ESCCs are predominated with six signatures (Fig. S2b): APOBEC (signature 2 and 13), dMMR (defective DNA mismatch repair, signature 6 and 15), BRCAness (signature 3), aging (signature 1), and drinking-related signatures (signature 16), which is in line with previous genomic studies on ESCCs [9,15,16]. Of three hypermutated patients (Supplementary Data 3c), two were MSI (microsatellite instability) patients with dMMR signature, while another was predominated with *APOBEC*-mediated signature (Fig. S2c). The aggregation of drinking habits and *ALDH2* variants increase the contribution of signature 16 (Fig. S2d). The *APOBEC*-mediated signature was associated with a high mutation burden in the cohort (*P* < 0.001, Spearman's r = 0.58, Fig. S2e), suggesting it was probably a mutator during ESCC carcinogenesis.

Somatic mutations in MRS can be classified as the trunk, branched, and private, respectively (detailed in Supplementary Data 3a). Trunk mutations refer to mutations present in all samples of the same patient. Branched mutations refer to mutations present in at least two samples but not all samples. Private mutations refer to mutations specific to one region. Distinct mutations might represent subclones of different clades during tumor evolution. An average of 56.9% of somatic mutations was heterogeneous (branched or private, Fig. S3a), suggesting extensive genetic intra-patient heterogeneity (gITH). Approximately 31%, 24%, and 45% of non-synonymous mutations were the trunk, branched, and private mutations, respectively, whereas these values are 53%, 17%, and 29% for well-known driver genes. We found clear evidence of positive selection (the ratio of non-synonymous to synonymous substitutions, also known as dN/dS) for nonsense and truncating mutations in ESCC-associated genes (EAG) set among the ESCC cohort (Fig. S3b), and dN/dS values were higher for the EAG set than for other genes (OG) set (Fig. S3c), confirming that the EAG set was enriched for true ESCC drivers. In comparison with the OG set, there is a significant correlation between the dN/dS ratio of the EAG set and the median CCF (cancer cell fraction) of their mutations (Fig. S3d). Further, we focused on the top 50 genes from the EAG set that frequently occur in ESCC, where their dN/dS ratio was more significantly correlated with the median CCF of their mutations (*P* < 0.001, Spearman's r = 0.45, Fig. S3e). These results suggest that early-occurring driver mutations usually confer stronger selective advantages. The burden of ESCC driver mutations at the patient level by MRS was higher than that from the single-site sequencing (Fig. S3f). For instance, the mutational prevalence of *NOTCH1* (35%) and *KMT2D* at the patient level was more than two times higher than that at the single-sample level (Fig. S3f). We noticed that many *NOTCH1* mutations were branched or private, which explains the significant differences in its prevalence between sample- and patient-level. We also noticed that *NOTCH1* is frequently mutated in normal epithelia of the esophagus [17,18], and *NOTCH1* mutations drive clonal expansion in the normal esophageal epithelium but impair tumor growth [19], suggesting the high prevalence of *NOTCH1*, to some extent, might originate from mutations of normal cells instead of tumor cells in our data.

As epigenetic changes also alter gene expression, we aggregated the somatic mutations with sample-level methylation to search for potential driver genes associated with ESCCs at the patient level (Supplementary Notes). Unexpectedly, most of the well-established driver genes seem unaffected by epigenetic changes. However, alterations in *PREX2* were observed in 23 out of 103 patients, including hyper-methylation of promoter regions in 15 patients (Fig. 1c, d, Fig. S4a), which had not been reported in ESCC previously. *PREX2* is a frequently mutated guanine nucleotide exchanger (GEF) in cancer, which interacts with the tumor suppressor *PTEN* to promote cell proliferation in colorectal, kidney, lung, and hepatocellular cancers [20,21]. But its role in ESCC remains elusive. In our cohort, the expression of *PREX2* was downregulated in tumors as compared to adjacent normal tissues and was negatively associated with methylation beta value (Fig. S4b, c), suggesting *PREX2* may be a tumor suppressor in ESCC. Furthermore, we found 15 *PREX2* mutations in the TCGA and previous Chinese and Japanese cohorts [22] (Fig. S4e). *PREX2* alterations were more frequent in female patients (*P* *=* 0.05, Fig. S4d). As *PREX2* has higher expression in the KYSE450 cell line compared to other esophagus cell lines (Fig. 1e), we chose the KYSE450 cell line for the knockdown experiment (Fig. 1f). The transwell assay and wound healing assay showed that compared with the control group, the KYSE450 cells of *PREX2* knockdown significantly increased the migration and invasion capacity of ESCC cells (Fig. 1g). All these results suggested that *PREX2* is a putative novel suppressor for ESCC.

### Molecular timing of driver alterations and phylogenetic analysis of ESCC evolution

**Figure 2.**
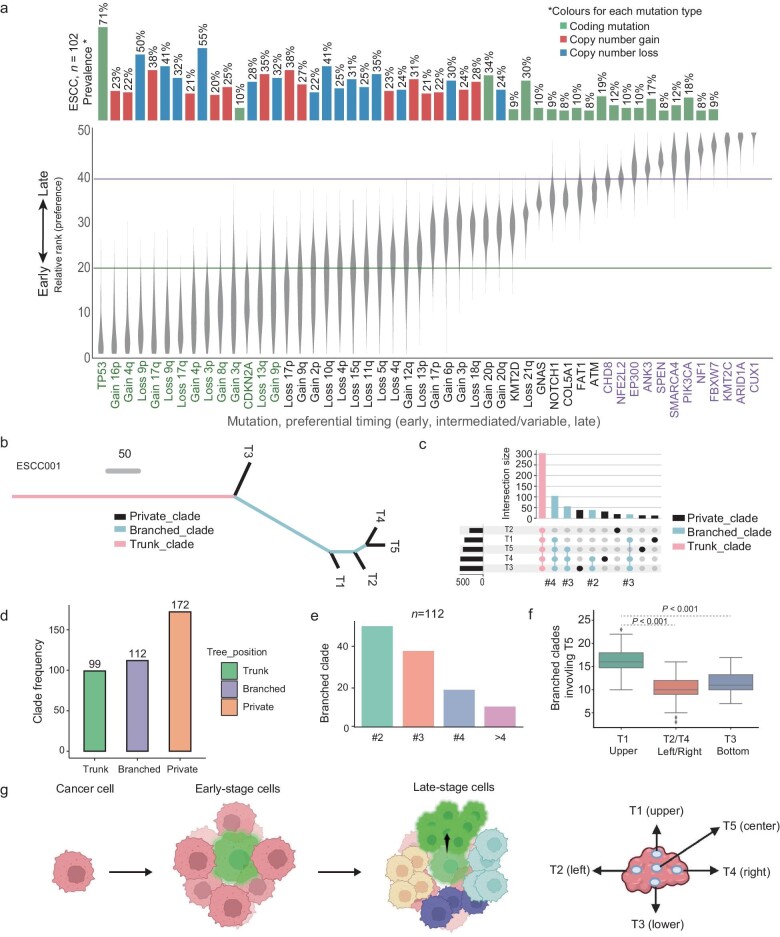
Genetic evolution underlying ESCC progression. (a) Molecular timing quantification of genetic mutations and chromosomal changes for ESCC. Mutations that occur in the early stage tend to have low relative rank values (events with odds below 20). The prevalence of each event type across 102 individuals is displayed as a bar plot on the top. (b and c) An example of a patient's phylogenetic tree in (b) and (c). Mutations of different stages are stratified into distinct clades. ‘#2’ represents a branched clade consisting of two regions. (d) The frequency of trunk, branched, and private clades across 103 patients. (e) The frequency of branched clades involving different tumor regions. In detail, ‘#2’ represents the clade consisting of two tumor regions. (f) The number of branched clades involving tumor center (T5) and marginal regions (T1–T4). The *p*-value is given based on the bootstrapping result (*n* = 100). Statistical analysis is performed with the Student's *t*-test. (g) A proposed model of subclonal evolution from normal to late-stage cancer cells. Different colors represent subclonal cells of tumors at different stages.

We further quantified the molecular timing for mutations or chromosomal alterations using phylogicNDT [23]. Overall, driver mutations of *TP53* and *CDKN2A* tended to occur early while mutations such as *NFE2L2, SMARCA4, PIK3CA*, and *CUX1* tended to occur in the relatively late stage. In contrast, the majority of chromosomal alterations tend to occur in the early or intermediated stage, suggesting that the karyotype transition was an early genomic event that might associate with tumor malignant transformation (Fig. 2a).

To investigate the geographical patterns of subclonal expansion, a phylogenetic tree was constructed using the parsimony maximum algorithm. The phylogenetic clade could be classified into the trunk, branched, and private clade, respectively (Fig. 2b, c, Supplementary Data 4). Genetic ITH (gITH) varied among patients (Fig. 1c, Supplementary Data 3b). High-gITH ESCCs demonstrate shorter trunk clades and longer branched or private clades in contrast to that with low gITH (Fig. S5). A total number of 99 trunk, 112 branched, and 172 private clades were identified (Fig. 2d, Supplementary Data 4), of which branched clades were further divided into clades involving two, three, and at least four tumor regions (Fig. 2e). The tumor center (T5) involved a high number of branched clades and a low number of private clades compared to marginal regions (Fig. S6a, b). Specifically, 63% of branched clades consisted of tumor centers, higher than that in marginal regions. The proportion of branched clades involving the marginal regions is comparable (upper: 50%; left: 48%; bottom: 53.6%; right: 44%). This finding was consistent with the model that subclones likely arise in the tumor center and expand from the center to the marginal regions (peripheral growth). This model was also supported by two findings: 1. Analyses of branched clades involving two regions revealed that the majority of these branched clades were between adjacent regions or between the marginal region and tumor center (Fig. S7a); 2. Analyses of branched clades involving three regions revealed the same trend that most of these subclones were derived from the tumor center (Fig. S7b).

We particularly focused on branched clades, as inform subclonal expansion across different regions. To investigate subclonal expansion over space, we mapped branched clades to physical regions of the primary tumors. The frequency of branched clades shared between the tumor center and marginal regions was examined. Interestingly, the upper region significantly harbors more branched clades with the tumor center than other regions (Fig. 2f), which indicated that subclones from the tumor center were more likely to expand upwards along the esophagus. Thus, we proposed a spatially directed evolutionary model for ESCC, during which subclones from the tumor center tend to expand upwards along the esophagus (Fig. 2g). In addition, 19 out of 103 (18.4%) ESCCs exhibit early LN dissemination, while 10 cases (19.3%) show late dissemination, indicating the prevalence of early and late LN metastasis was comparable in ESCCs. Inspection of ESCCs with multiple LN metastases revealed that linear metastasis was the predominant pattern in ESCCs, and a few ESCCs show metastasis-metastasis pattern that metastatic subclone spread from metastasis to another metastasis (Fig. S8).

### Intra- and inter-patient heterogeneity of tumor microenvironment

To investigate the heterogeneity of the tumor microenvironment, we first employed MethylCIBERSORT [24] on 460 primary tumors of 103 ESCC patients with genome-wide DNA methylation data to deconvolute the cellular components in each region (Supplementary Data 5). This method could help identify 11 cell types within a tumor, such as immune and stromal cells. To evaluate the accuracy of the deconvolution for each type, we compared the cancer fraction predicted from CIBERSORT to that from whole-exome sequencing data. The values predicted from CIBERSORT were strongly associated with the purity of the samples predicted by ABSOLUTE (*P* < 0.0001, Spearman's r = 0.7, Fig. S9), indicating that deconvolution was adequate from methylation data.

We found significant correlations between multiple cell types in line with similar patterns in other human cancers [24]. Specifically, the abundance of CD8^+^ T cells positively correlates with B cells (Fig. S10a). It was negatively correlated with natural killer cells. Endothelial cells are associated with fibroblast and are inversely correlated with CD8^+^ T cell abundance. We also observed a significant correlation between somatic mutation burden and CD8^+^ T cells (*P* < 0.0001, Spearman's r = 0.38) and stromal cell abundance (*P* < 0.0001, Spearman's r = −0.42; Fig. S10b). Cell estimates displayed overall intra-tumor and inter-patient heterogeneity in cell components (Fig. S11a). Moreover, high heterogeneity was observed for different immune cell types (Fig. S11b). Neutrophils, cytotoxic T lymphocytes, and B cells showed the most regional variations (Fig. S11c). A high proportion of cytotoxic T lymphocytes (≥10%) are present in 38% (174 out of 460) regions.

**Figure 3.**
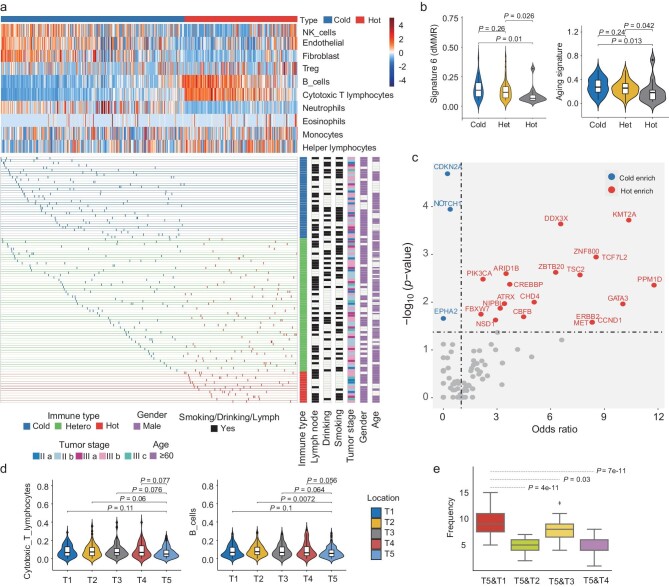
The heterogeneity of immune microenvironment. (a) Unsupervised clustering of cell components in tumor regions (Manhattan Distance). The upper panel shows the abundance for each cell type and two clusters; the bottom panel shows the distribution of regional tumors in each patient, and the clinal information is shown on the right. (b) Box plots display the association of mutation signatures with hot/cold tumor microenvironment. (c) The dot plot displays the association of mutagenesis with the hot/cold tumor microenvironment. (d) Violin plot of the differential abundance of cell types between marginal regions and tumor center. (e) The frequency of minimal-immune distance pair across ESCC patients. The *P*-value is given by bootstrapping results (*n* = 100).

As heterogeneity was widely present across different cell types, we further classified each tumor region (*n* = 460) into immune-cold and immune-hot tumors by unsupervised clustering (Fig. 3a). Hot and cold tumors were respectively characterized by a high and low percentage of B cells, CD8^+^ T cells, and CD4^+^ helper/effector T-lymphocytes (Fig. 3a). Multiple immune-associated genes such as *CD79A, CD3E, CD3D, ZAP70, CXCR6*, and *GZMA* were upregulated in hot tumor regions. Conversely, ECM-related genes were highly expressed in cold tumor regions (Fig. S12a). As expected, hot tumor regions displayed markedly higher anti-PD1 favor scores (Fig. S12b), higher expression, and lower methylation for genes *CD274* and *CTLA4* (Fig. S12c and Supplementary Data 5). Further, by analyzing immune repertoire sequencing data of 10 ESCC patients subjected to TCR sequencing previously [9], we found that unique clones from both TCR-alpha and -beta were significantly elevated in hot than cold tumor regions (Fig. S12d). Altogether, these results suggest our immune subtyping was adequate.

At the whole-tumor level, hot, cold, or heterogeneous tumors were identified based on whether multiple regions consistently show immune-hot or immune-cold characteristics. There were 34 cold, 56 heterogeneous, and 13 hot primary tumors, respectively (detailed in Supplementary Data 3b). Heterogeneous tumors comprised both hot and cold regions. The overall mutation burden at the patient level in heterogeneous/hot patients was higher than in cold patients (*P* = 0.002, Fig. S12e; Supplementary Data 3b). Interestingly, aging and dMMR mutational signatures tend to decrease in hot patients (Fig. 3b and Supplementary Data 3c). Next, we also found that the patients with drinking habits tended to enrich fewer CD8^+^ T cells (*P* < 0.01, Fig. S13a; Supplementary Data 1 and 5). As mutational signature 16 is associated with drinking habits [16], we inspected the proportion of CD8^+^ T cells in patients with high and low signature 16, respectively. Indeed, the data showed that patients with low signature 16 harbored higher CD8^+^ T cells than their counterparts (Fig. S13a, Supplementary Data 3c and 5).

We next assessed the genomic alterations that associate with particular immune clusters. *CDKN2A, NOTCH1*, and *EPHA2* mutations were enriched in cold tumors, while *PIK3CA, ERBB2*, and *CCND1* mutations were increased in hot tumors (Fig. 3c; Supplementary Data 3b and 3d). To validate the interaction of these gene variations with the immune microenvironment in ESCC, TCGA ESCC methylation data was reanalyzed which included 95 samples. These ESCCs were clustered into 39 cold and 56 hot tumors, respectively, showing the same differential trend among immune systems cell compositions (Fig. S13b). Interestingly, we also observed the enrichment of *CDKN2A* and *NOTCH1* mutations in cold tumors, and *ERBB2* mutations in hot tumors (Fig. S13b), indicating these driver gene mutations play an important role in immune infiltration. Subclonal somatic copy number alterations (CNAs) were also associated with the immune microenvironment, where cold ESCCs possessed numerous clonal chromosomal-arm changes (Fig. S14a, b; Supplementary Data 3b). The correlation between CD8^+^ T abundance and CNA ITH was not significant in cold tumors (*P* = 0.75), but significant in hot tumors (*P* = 0.03), suggesting a hot microenvironment promotes genetic ITH (Fig. S14c, d; Supplementary Data 5 and 7).

Next, we compared the cytotoxic cell infiltrates and B cells in different locations and found that the tumor center had a lower abundance than tumor margins (Fig. 3d). This data was consistent with the previous finding in other human cancers where immune cells at the tumor center are less abundant than that in the tumor margin [25]. The abundance of immune cells did not show consistent differences in any two locations from the tumor margin, suggesting that immune infiltrates were nearly uniformly present in marginal regions (Fig. S13c). We employed a bootstrapping method (*n* = 100) to evaluate the frequency of one patient's most minimal immune distance between the tumor center and marginal regions. Interestingly, the upper region exhibits significantly higher similarities with the tumor center (Fig. 3e). This finding was consistent with the spatially directed evolutionary model of ESCCs where subclones derived from the tumor center have a preferential expansion upwards along the esophagus (Fig. 2g).

### Immunoediting depends on the immune context and is associated with prognosis in ESCCs

**Figure 4.**
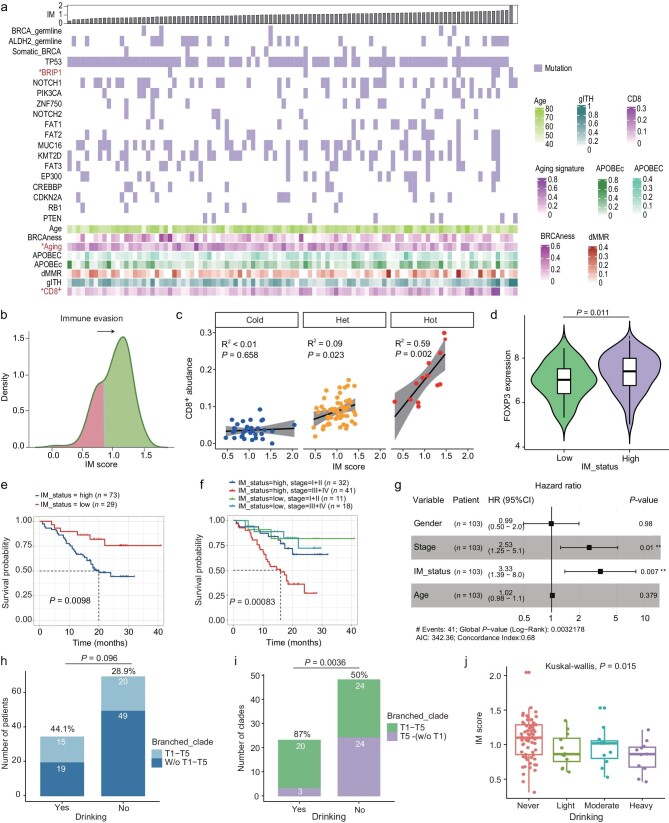
Immunoediting activity and its impact on clinical metrics across 103 patients. (a) The association between immunoediting (IM) score with genetic mutations. The significance of a *P*-value less than 0.05 is marked with a red star. (b) The density of immunoediting score shows two modes, which refer to low (low IM score) and high (high IM score) immune evasion. (c) The correlation between CD8^+^ T cell abundance with immunoediting score in cold/het/hot patients. (d) The expression of gene *FOXP3* in hot patients of low and high IM scores. The two-group test is based on the Wilcoxon test. (e) Survival curves of tumors with IM_status_high (*n* = 73) against IM_status_low (*n* = 29). Statistical analysis is performed with a Log-rank test. (f) The impact of IM status and TNM stage on overall survival. Statistical analysis is performed with a Log-rank test. (g) The impact of IM status on the overall survival of ESCC patients was adjusted by sex, age, and TMN stage. *P ≤ 0.05, **P ≤ 0.01. (h) The number of patients harbored branched clades involving T1 and T5 (one-sided Fisher's exact test). (i) The number of branched clades involving T5 and T1/other regions (w/o: without, two-sided Fisher's exact test). (j) The distribution of IM scores across four drinking groups.

To further explore the impact of immune-tumor interaction on ESCC, we analyzed the immunoediting capacity for the trunk and heterogeneous (including branched and private) mutations. The immunoediting (IM) score (Supplementary Notes) of distinct clades indicated strong and weak immune evasion (Fig. 4a, b; Supplementary Data 3b). Strong immunoediting (low immunoediting score) means that neoantigens could be eliminated by the immune system leading to a reduced observed neoantigen. We compared the immunoediting activity of 103 patients through the IM score of trunk clades (Supplementary Data 4). Drinking habits, *BRIP1* mutations, and aging signature each show an association with IM score (*P* < 0.05, Fig. 4a and j). Interestingly, immunoediting activity strongly depended on CD8^+^ T cell level (Fig. 4c). The correlation between immunoediting activity and CD8^+^ T cell estimates soared with the increase of CD8^+^ T cell abundance. Specifically, the IM score was not associated with CD8^+^ T cell estimates in immune cold tumors, whereas the IM score positively correlates with CD8^+^ T estimates in hot tumors (*P* = 0.002, R^2^ = 0.59, Fig. 4c), indicating more immune infiltrated ESCCs evolved higher immune evasion. This result also revealed a strong immunosuppressive nature in hot tumors of ESCCs. As *FOXP3* is a master regulator that turns the immune response down, we thus looked at its expression in hot patients. The expression of *FOXP3* in the patients with high IM scores was higher than that with low IM scores in the hot patients (*P* = 0.01, Fig. 4d). These results suggest that ESCCs seem more likely to undergo a process from immunosurveillance to immune evasion in the hot patient. These findings help explain the high genetic heterogeneity within the hot microenvironment.

We also examined spatial immunoediting across distinct phylogenetic clades (Fig. 2b). Notably, branched clade involving the upper region of primary tumors showed significantly stronger immunoediting than subclones involved in other regions (*P* = 0.03, Fig. S15b; Supplementary Data 4). Subclones involved in LN metastases tend to harbor weak immunoediting activity despite not achieving significance, indicating immune evasion in LN metastasis. This result was consistent with our proposal that ESCC undergoes spatially directed evolution. As immune evasion was profound in hot ESCCs, we are wondering whether immune-related metrics could predict the clinical outcome. In our data, neoantigen burdens were significantly elevated in hot tumors (Fig. S16a), and it positively correlates with CD8^+^ T cell composition (Fig. S16b). These trends persisted in both clonal and subclonal neoantigens. However, we did not find associations between clinical outcomes and neoantigen burden or CD8^+^ T cells (Fig. S16c). These patterns were verified by the datasets of TCGA [6] methylation, RNA-seq, and GSE53624 [26] microarray expression in ESCC (Fig. S16d), suggesting these two indicators were not prognosis factors in ESCC. In contrast, the abundance of stromal cells was moderately associated with poor outcomes, consistent with an earlier report [27]. Interestingly, we found that patients with strong immunoediting exhibited better overall survival than those with weak immunoediting (*P* = 0.0098, Fig. 4e). Together with the TNM stage, it better predicted patients’ outcomes (*P* = 0.00083, Fig. 4f). When adjusted with TNM stage, gender, and age, the IM score was an independent prognostic factor (Fig. 4g). These results suggest that the IM score, instead of mutation burden and CD8^+^ T cell abundance, could be a valuable predictor for ESCC outcomes.

As the spatially directed evolution is supported by several lines of evidence including multi-regional phylogenetic trees, subclonal expansions, and immunoediting profiles, we anticipated that directed evolution might be related to differential stimulation from exogenous food or drinking, but evidence was lacking. We noticed that our 103 patients have information on alcohol drinking and tobacco smoking, thus we wondered whether drinking and smoking habits could be associated with directed evolution. Interestingly, we found that the proportion of patients with T1 (upper) and T5 (center) in the same branched clade is much higher in the drinking group as compared to the non-drinking group (44.1% vs. 28.9%, Fig. 4h). This indicated that branched clade is more likely present in ESCC patients who have drinking habits. Further, we specifically inspected the number of branched clades involving T5, and we found that 20 out of 23 branched clades involved T1 and T5 in drinkers, which is significantly higher than that in non-drinker ESCCs (*P* = 0.003, Fig. 4i). These results suggest that drinking might drive the subclone within the tumor center to more likely expand upwards in ESCCs. However, we did not find a similar pattern in ESCC with smoking (Fig. S15a). These results also pinpoint that drinking is probably one of the mechanisms for spatially directed evolution, which is consistent with our hypothesis that directed evolution is probably caused by the differential stimulation from exogenous food. In addition, ESCCs with drinking habits tend to have strong immunoediting (Fig. 4j), which explains why the branched clade of the upper region undergoes stronger immunoediting (Fig. S15b). We also inspected spatial differences of different anatomical locations such as middle, lower, and upper, and we found that directed evolution is more pronounced in ESCCs of upper/middle esophagus (Fig. S17).

### The immune microenvironment dictates varying selection pressure within the tumor

**Figure 5.**
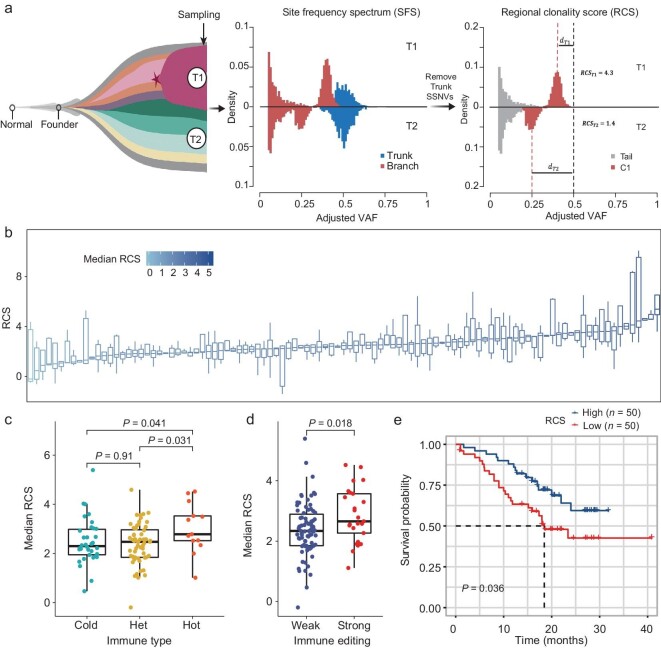
Selective pressure was affected by the tumor microenvironment. (a) Schematic overview of regional clonality score (RCS) computational framework for tumor expansion via a stochastic branching process followed by spatial sampling and multi-regional sequencing (Supplementary Notes). (b) The distribution of RCS values of each tumor. The color gradations within the blue color represent the contributions of the median RCS of each tumor. (c and d) Boxplots illustrate the probability density of median RCS values amongst tumors with distinct immune types (‘Cold’, *n* = 34; ‘Het’, *n* = 56; ‘Hot’, *n* = 13) or tumors with strong (*n* = 78) and weak (*n* = 25) immune evasion, respectively. *P*-value, two-sided Wilcoxon Rank-Sum Test. (e) Survival curves of tumors with high RCS (*n* = 50) against low RCS (*n* = 50). Statistical analysis is performed with a Log-rank test.

Previously, we showed evidence of selection during tumor growth based on MRS data analysis in a small ESCC cohort [9]. This evolutionary model was now validated by the finding that most primary tumors in this cohort (66 out of 103 ESCC tumors) were poorly fitted to the neutral evolution model by a statistical test for their site-frequency spectrum (SFS, also known as variant allele frequency (VAF) distributions [28]) (Supplementary Data 6; Supplementary Notes). For example, ESCC001 consisted of a clonal peak and a neutral tail of somatic mutations that were consistent with neutral evolution (Fig. S18a, b), while ESCC024 harbored a sub-clone with selective advantage (Fig. S18c) consistent with Darwinian selection [29] (Fig. S18d). Considering the expansion of subclonal mutations driven by selection during tumor growth, we defined the regional clonality score (RCS) to estimate the selective pressure of subclones based on the subclonal mutational cluster with the highest clonality (Fig. 5a; Supplementary Data 6 and 7; Supplementary Notes). Interestingly, ESCCs under strong selection exhibit significantly higher gITH (*P* < 0.001) and achieve a marginally significant increase for copy number ITH (*P* = 0.07) as compared to samples with neutral evolution. However, they did not show a significant difference for tITH and iITH (immune ITH) between selection and neutral evolution (Fig. S19a). As expected, tumors under selection carried fewer clonal non-silent mutations but higher subclonal non-silent mutations. The gITH and RCS scores were also higher in tumors under selection as compared to tumors following neutral evolution (Fig. S19b). In addition, the RCS values were positively associated with genetic ITH and the genetic distance (FST, Supplementary  Notes) within the tumor (Fig. S20). The variable RCS values of 461 primary samples from 103 ESCC patients (Supplementary Data 7), further supported pervasive subclonal selection in ESCC (Fig. 5b). High variation of the clonality scores within and between tumors also indicated uneven selection across tumor regions during tumor growth (Fig. 5b), in line with the high heterogeneity of within-tumor immune microenvironments. Interestingly, the upper-esophagus tumors showed a higher distance of the SFS within distinct regions (Fig. S21a), and they also exhibited more variations of RCS values (Fig. S21b). Meanwhile, the longitudinal genetic divergence (T1 vs. T3) within the tumor was the highest, followed by the transverse (T2 vs. T4) (Fig. S21c). In contrast, the tumor margin, especially the upper margin region (T1), also had a stronger expansion ability than the tumor center (Fig. S21d). This quantification of clonal expansions again supports the spatially directed evolutionary model (Fig. 2g).

To explore the impact of TME on selective pressure, we found that hot tumors have higher RCS than heterogeneous and cold tumors (Fig. 5c). Similarly, tumors with low immunoediting scores (strong immunoediting) had higher RCS values (Fig. 5d), which indicated that the tumor immune microenvironment facilitates the subclonal fitness and growth of tumor cells. Remarkably, patients with lower RCS tended to have a worse prognosis (Fig. 5e), probably related to weaker immunoediting in these tumors (Fig. 4c, Fig. 5d). These results elucidated the reciprocal signal between cancers and the surrounding immune microenvironments, supporting the fact that the TME is crucial for tumor development and progress, contributing to spatial intra-tumor heterogeneity [30].

### BRCAness ESCCs demonstrate a better outcome

**Figure 6.**
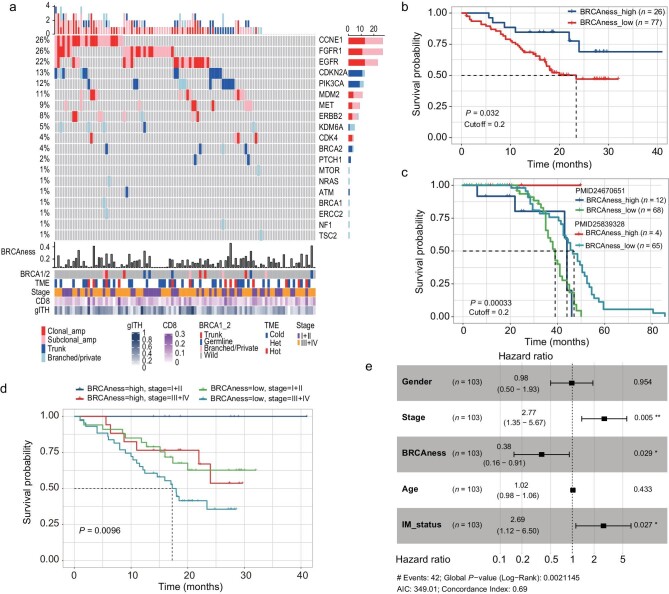
The landscape of actionable targets across 103 patients. (a) The distribution and heterogeneity of genomic targets curated in the OncoKB database. (b) The impact of BRCAness status on ESCCs’ overall survival. (c) The impact of BRCAness status on ESCCs’ outcome in validation cohorts (*n* = 149) is shown. (d) The impact of BRCAness and tumor stage on ESCCs’ outcome in study cohort. (e) The Cox-regression of ESCCs’ survival was adjusted by multiple variables. Statistical analysis is performed with a Log-rank test.

Finally, to investigate the heterogeneity of therapeutic targets, we collected the potential targets driven by mutations and amplifications curated in oncoKB [31]. A total of 19 targeted genes involving 152 actionable alterations were identified (Fig. 6a), of which 49.3% of alterations were heterogeneous. Prevalent amplified genes *CCNE1, FGFR1, EGFR, MDM2, MET*, and *ERBB2* exhibited high heterogeneity, accounting for ∼50% of the amplifications. In contrast, the majority of *CDKN2A* and *PIK3CA* mutations were trunk mutations (Supplementary Data 3d), suggesting the potential value as inhibitor of the PI3K/Akt/mTOR pathway. In addition, we noticed that 14 out of 103 patients (13.5%) harbor *BRCA1/2* germline or somatic variants. Of these 14 variants, there are five germline variants, five trunk mutations, and four heterogeneous mutations (Supplementary Data 3d). In line with our previous findings [9], patients with early variants (germline or trunk mutations) tended to harbor a high contribution of BRCAness signature (Fig. S22a). ESCCs with branched or private *BRCA1/2* mutations exhibit lower BRCAness signature than those without BRCA mutations.

 BRCAness patients tend to benefit from platinum-based therapy in prostate cancer and breast cancer [32,33]. As platinum-based chemotherapy is frequently used in ESCC for treatment before or after surgery, we were wondering whether patients with high BRCAness signatures could also benefit. Thus, we analyzed the overall survival in our data and found that BRCAness patients (contribution ≥0.2) demonstrated significant favorable outcomes (*P* = 0.032, Fig. 6b). We also quantified the HRD (homologous recombination deficiency) score for each genome using scarHRD [34] and found that ESCCs with a high HRD score tend to have a better prognosis (Fig. S22b). The same trend was evident in an independent validation cohort [32,35] with favorable outcomes in four patients with high BRCAness in Zhang's cohort [32] (Fig. 6c). BRCAness patients with a low TNM stage showed favorable outcomes compared to non-BRCAness patients with a high stage (*P* = 0.0096, Fig. 6d). Adjusted by age, gender, stage, and IM score, BRCAness could still predict good outcomes (Fig. 6e). These results suggest that high-BRCAness ESCCs might need a specific treatment strategy. In addition, we found *CDKN2A* mutations seem to be depleted in BRCAness ESCCs (Fig. S22a, c), indicating ESCCs with a high BRCAness signature might harbor distinct genomic profiles.

## DISCUSSION

In the past, the characterization of genetic mutations and evolution of ESCCs has been largely limited to single biopsy, single-omics, or small-size cohorts. Our study presented the latest multi-omic atlas (507 tumor samples from 103 ESCC patients) with a systematic evolutionary analysis of ESCCs. Our data revealed extensive spatial heterogeneity of genomic alterations, microenvironment components, and subclonal selection in ESCC. We highlighted an immunosuppressive nature within hot patients, which was associated with high immune evasion and high genetic heterogeneity. Based on the patterns of clonal expansion and microenvironmental characteristics, we proposed a model of spatially directed evolution within ESCCs. Importantly, we found that BRCAness ESCCs exhibit better outcomes in independent cohorts. As platinum-based chemotherapy is frequently used for ESCC treatment before or after surgery, BRCAness ESCCs might also get the benefit from platinum-based chemotherapies. However, future studies based on a well-controlled clinical trial are warranted to verify this hypothesis.

This study unveiled the spatial heterogeneity of ESCCs’ microenvironment and genetic mutations through multi-omics data. The prevalence of most driver genes is largely underestimated compared to single-sampling sequencing due to extensive subclonal mutations. By aggregation of genome and methylation, we found that most driver genes seem not likely to suffer methylation changes, which prompted us to posit that gene regulation from methylation is inherently different from somatic mutations. Despite this, we also nominated a novel suppressor, *PREX2*, which interacts with the tumor suppressor *PTEN* to promote cell proliferation. One of the strategies targeting *PTEN* to restrain tumor cell invasion is to inhibit *PREX2*-catalyzed activation [36], which suggests that drugs targeting *PTEN* might need to consider the activity of *PREX2*. In addition, Mai *et al.* reported that *PREX2* mutations were enriched in low-risk patients [37], which is not consistent with our study, indicating the genetic and phenotype heterogeneity across different cohorts.

We propose an evolutionary model, termed spatially directed evolution, for ESCC diversification. First, the frequency of shared branched clades between the tumor center and upper region is significantly higher than that between the tumor center and other marginal regions, suggesting that subclones originating from the tumor center tend to grow toward the upper side of the tumor mass. Second, the minimal distance of cell component between the tumor center and marginal regions are more frequent between the tumor center and upper region. Third, subclones involved in upper regions tend to have higher IM scores compared to subclones in other regions. In contrast, these variations do not exist between the left and right sides of the primary tumors. These findings suggest that clonal diversification in ESCC is not stochastic in direction, and it is more likely to undergo a directed evolutionary pattern. Directed evolution is probably owing to differential stimulation from exogenous food. For instance, ESCC patients suffer difficulty in swallowing because of a tumor mass in the esophagus. When digestive food flows from the esophagus to the stomach, tumor cells in the upper region of the tumor mass seem much easier to directly come into contact with the exogenous food. In addition, esophagus peristalsis is another possible cause of spatially directed evolution. However, because of the complex interactions between esophagus tissues and external stimuli, it's difficult to pinpoint the exact mechanism of spatially directed evolution observed in our current study.

Although 80% of ESCCs carry actionable alterations, ∼50% of them are heterogeneous. These partially implicate the failure of targeted therapy for ESCCs [38]. It seems necessary to evaluate the clonal status of actionable targets before targeted therapy. In addition, BRCAness could stratify ESCC patients into different outcomes, indicating the positive impact of BRCAness on platinum-based therapy. Accurate and rapid identification of BRCAness is confounded by the economic burden of genome sequencing. Using germline or somatic mutations *BRCA1/2* is the only alternative approach for BRCAness identification. However, it might overestimate or underestimate the BRCAness by misleading the subclonal mutations or other alterations such as epigenetic changes. Recently, Tristan *et al.* employed deep learning to identify homologous recombination DNA-repair deficiency (HRD) through whole slide imuUages [39], which provides the possibility to identify BRCAness from stained tissue slides instead of sequencing.

In summary, this study provides comprehensive multilayer heterogeneity in ESCC through multi-regional omics investigations. We propose a new spatially directed evolutionary model and emphasize the importance of focusing on spatial heterogeneity in designing more efficient targeted therapies for ESCC.

## MATERIALS AND METHODS

For detailed materials and methods, please see the Supplementary Notes. The study protocol for human specimen collection was approved by the ethical committees of Shanxi Medical University. Written informed consents were obtained from all subjects.

## Supplementary Material

nwae150_Supplemental_Files

## Data Availability

The raw sequencing data generated in this study have been deposited in the Genome Sequence Archive (Genomics, Proteomics & Bioinformatics 2021) in the National Genomics Data Center (Nucleic Acids Res 2022), China National Center for Bioinformation/Beijing Institute of Genomics, Chinese Academy of Sciences. GSA-Human: https://ngdc.cncb.ac.cn/gsa-human/browse/HRA005046 (RNA-seq and WES); Zenodo: https://zenodo.org/records/11124261 (850 K array profiling).

## CODE AVAILABILITY

We have made the code available (https://github.com/ZHOUYong0530/ESCC-mutl2022).
