# Supplementary material for: Immune-tumor interaction dictates spatially directed evolution of esophageal squamous cell carcinoma
Source: Natl Sci Rev. 2024 Apr 23;11(5):nwae150. doi: 10.1093/nsr/nwae150 (PMC11129594; doi:10.1093/nsr/nwae150)
Supplement: nwae150_Supplemental_Files [file nwae150_supplemental_files.zip › Supplementary Figures.docx]

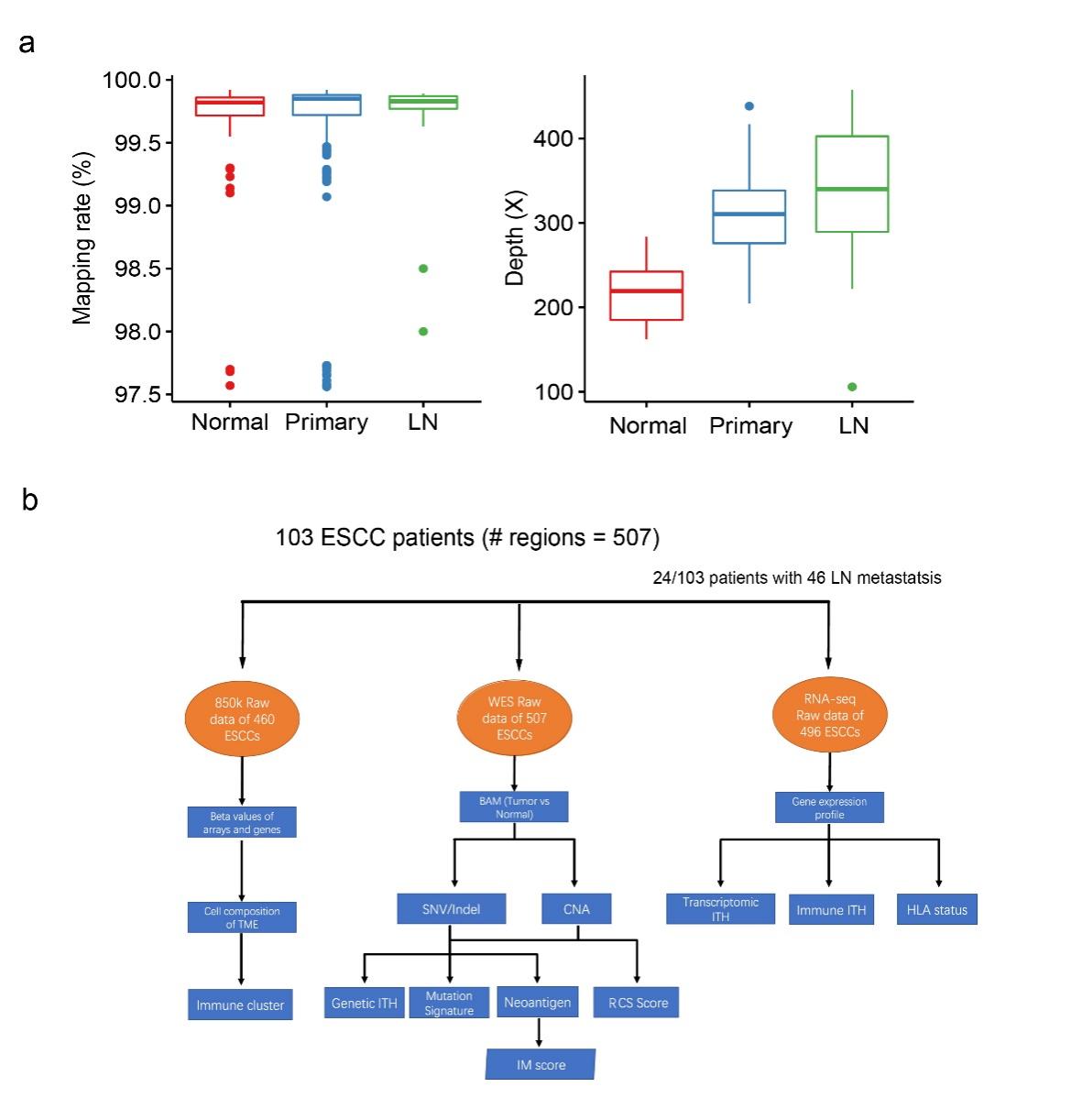


**Supplementary Figure 1. ESCC multi-omics sequencing. (a)** The mapping rate and sequencing depth of WES data among adjacent normal tissues, primary tumors, and lymph-node metastasis. Box plot data are presented as the median ± standard deviation. **(b)** Flow chart of bioinformatic analysis for spatial multi-omics.


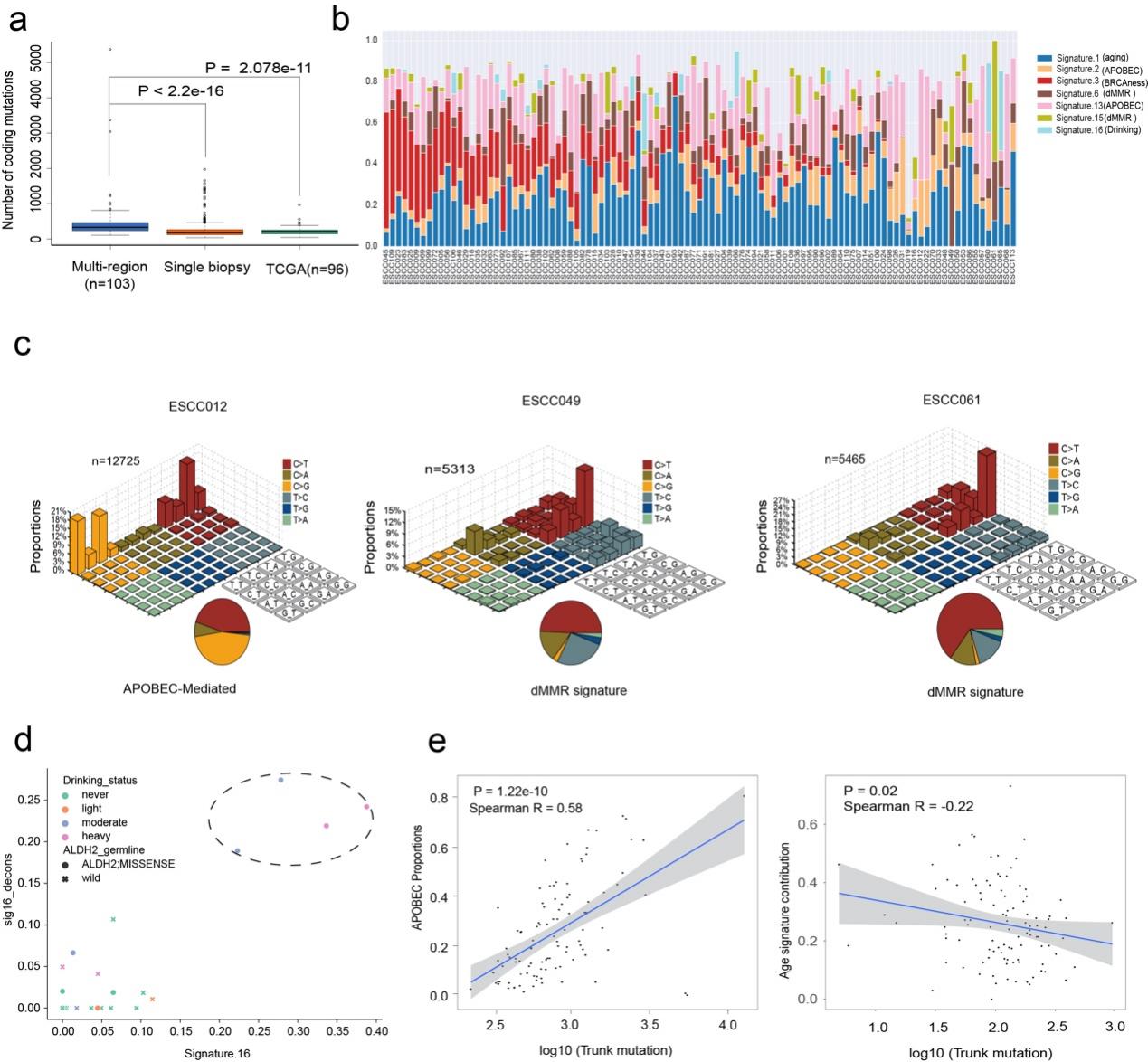


**Supplementary Figure 2. ESCC somatic mutations. (a)** Boxplot of coding mutation burden for three ESCC cohorts. Box plot data are presented as the median ± standard deviation. On the boxplots, the horizontal line indicates the median, the box indicates the first to third quartile and the whiskers indicate 1.5 × the interquartile range. The two-group test is based on the Wilcoxon test. **(b)** The contribution of 6 predominant mutational signatures operative in each patient. **(c)** Mutational signature of three hyper-mutated patients, including pie and 3D bar plots. **(d)** The contribution of signature16 was shown by Deconstructsig (y-axis) and MutationalPattern (x-axis). **(e)** The scatter plot indicated the relationship between trunk mutations and APOBEC signature (left), as well as between trunk mutations and aging signature (right). Spearman’s correlation and P value are reported. The line indicates the linear regression and the gray shading indicates the 95% CI of the regression.


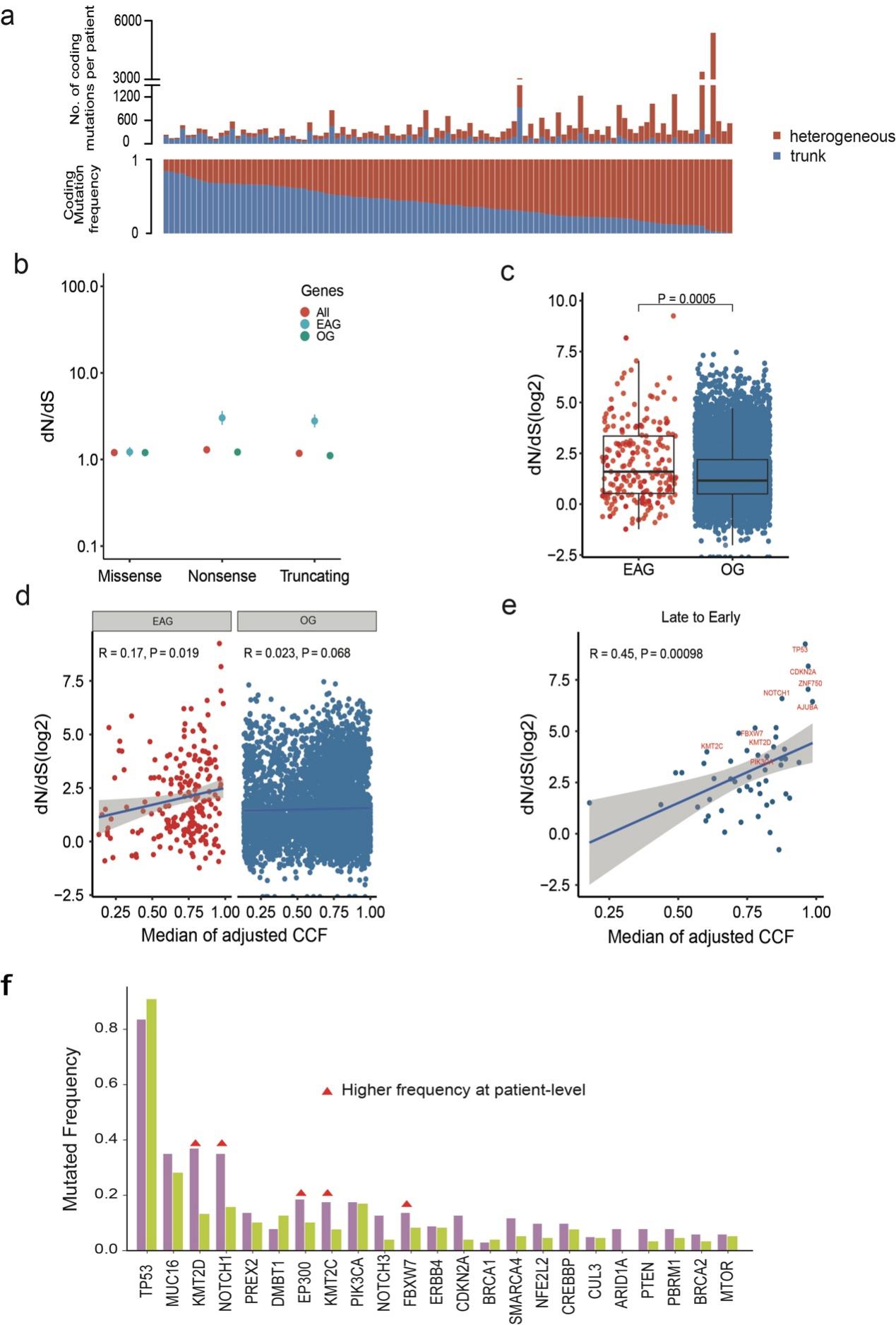


**Supplementary Figure 3. Multi-region sequencing reveals heterogeneous mutations in ESCC. (a)** Mutation burden of trunk (red) and heterogeneous (blue) mutations. **(b)** The global dN/dS values for missense, nonsense, and truncating substitutions for the EAG (ESCC-associated gene) set and OG (other gene) set across the ESCC cohort. Error bars indicate 95% confidence intervals. **(c)** Comparison of per-gene dN/dS value for EAG set and OG set. P value, two-sided Wilcoxon rank-sum test. **(d, e)** The correlation between per-gene dN/dS value and CCF for the EAG set, OG set **(d)**, and top 50 EAGs **(e)** with high frequency in the ESCC cohort, respectively. Spearman’s correlation and P value are reported. The line indicates the linear regression and the gray shading indicates the 95% CI of the regression. **(f)** Bar-plot of the mutation burden of ESCC driver genes at the patient level and the sample level. Purple represented the patient level, and yellow represented the sample level.


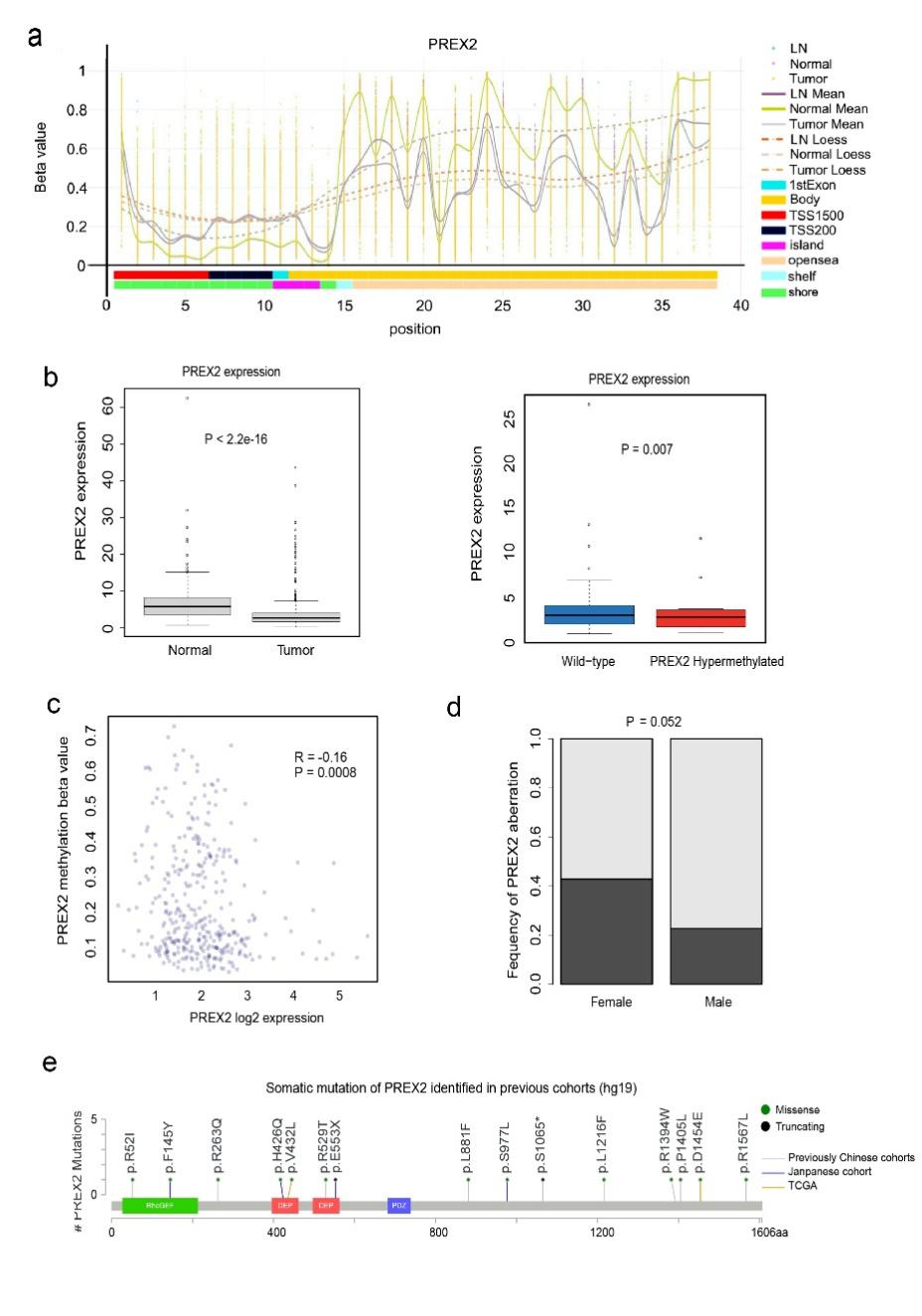


**Supplementary Figure 4. Driver gene *PREX2*. (a)** Hyper-methylation distribution of *PREX2* in ESCCs. **(b)** Left: Box-plot showed the expression of *PREX2* in normal and tumor samples. Right: Box-plot showed the expression of *PREX2* in hyper-methylated and wild-type samples. Statistical analysis is performed with the student-t test. **(c)** Scatter-plot of the correlation (spearman) between *PREX2* expression and *PREX2* methylation. **(d)** The stacked bar plot showed the proportion of *PREX2* aberration in different genders. **(e)** Mutation distribution of *PREX2* in other cohorts. The two-group test is based on the Wilcoxon test.


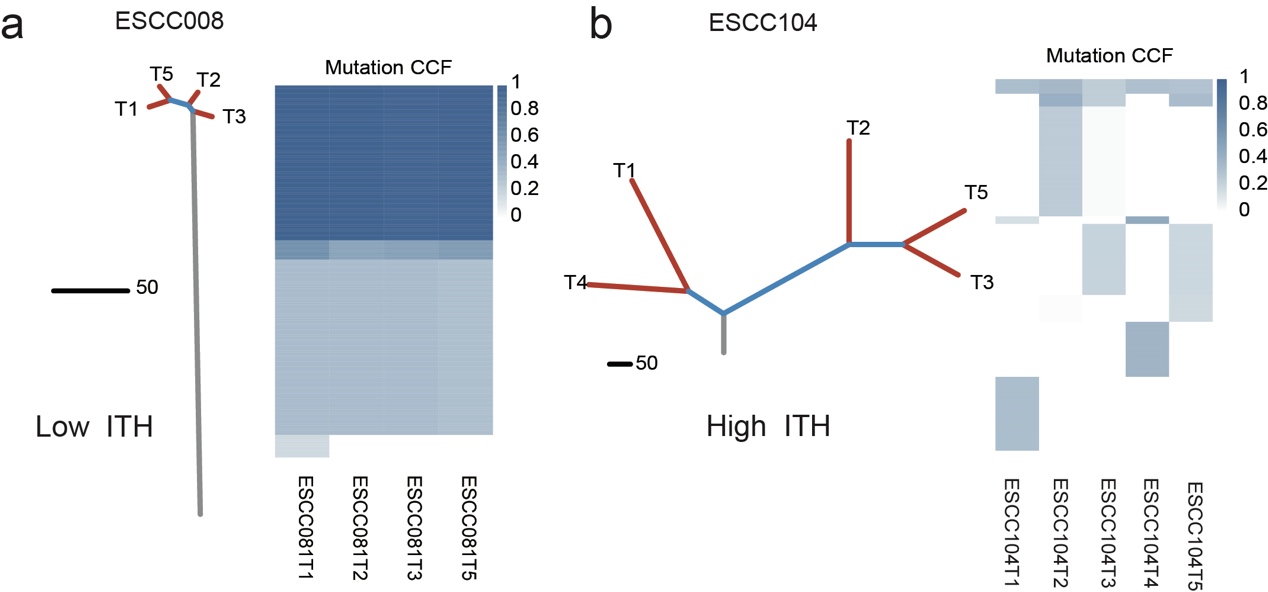


**Supplementary Figure 5. Evolutionary tree of two ESCCs with low and high gITH.** Gray represents the trunk, blue represents the branch, and red represents the private in the tree. Heatmap showing the regional distribution of somatic mutations based on their CCF in matched case.


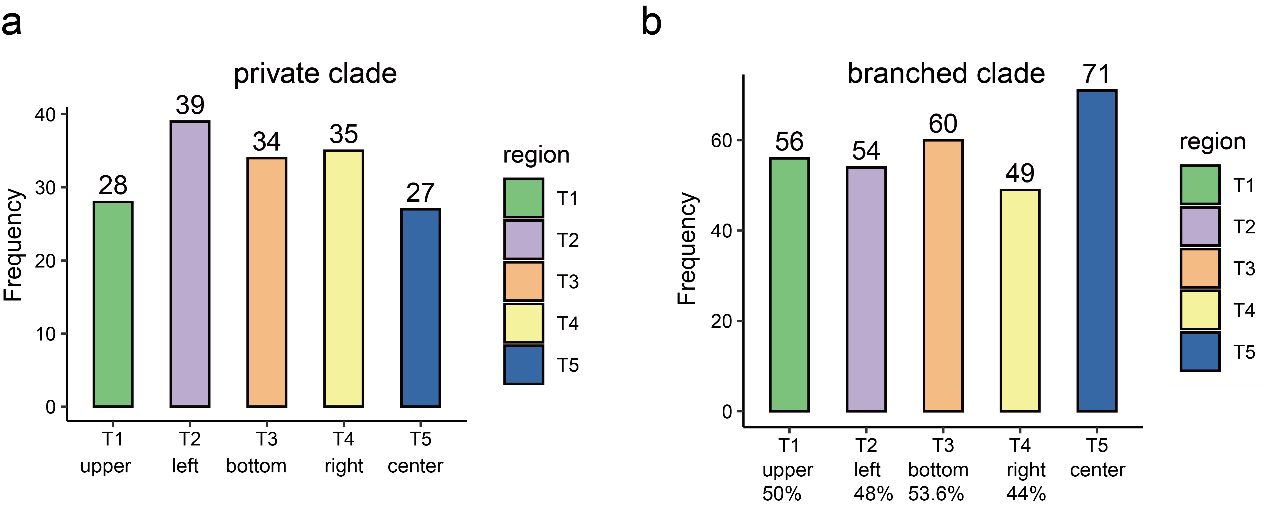


**Supplementary Figure 6. Genomic ITH of ESCC. (a)** The bar plot showed the frequency of private clades at different sub-regions (color) of ESCC. **(b)** The bar plot showed the frequency of branched clades at different sub-regions (color) of ESCC.


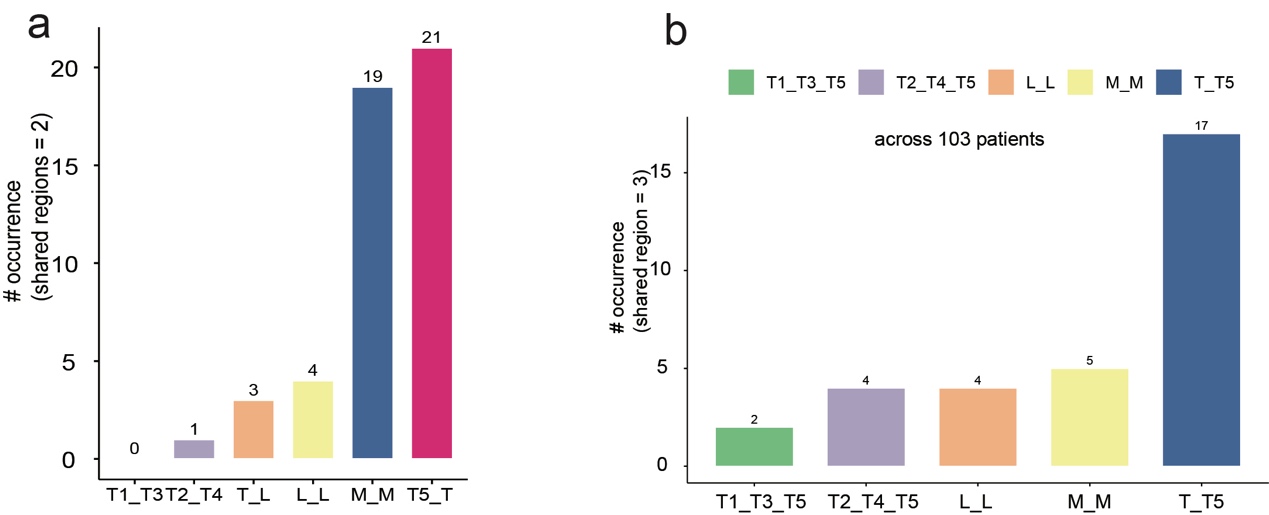


**Supplementary Figure 7. Shared clades between different regions. (a)** The bar plot showed the number of shared branch clades between two different regions. **(b)** The bar plot showed the number of shared branch clades in three different regions. T represents the sub-region in the primary tumor, L represents LN metastasis, and M represents the margin region in the primary tumor.


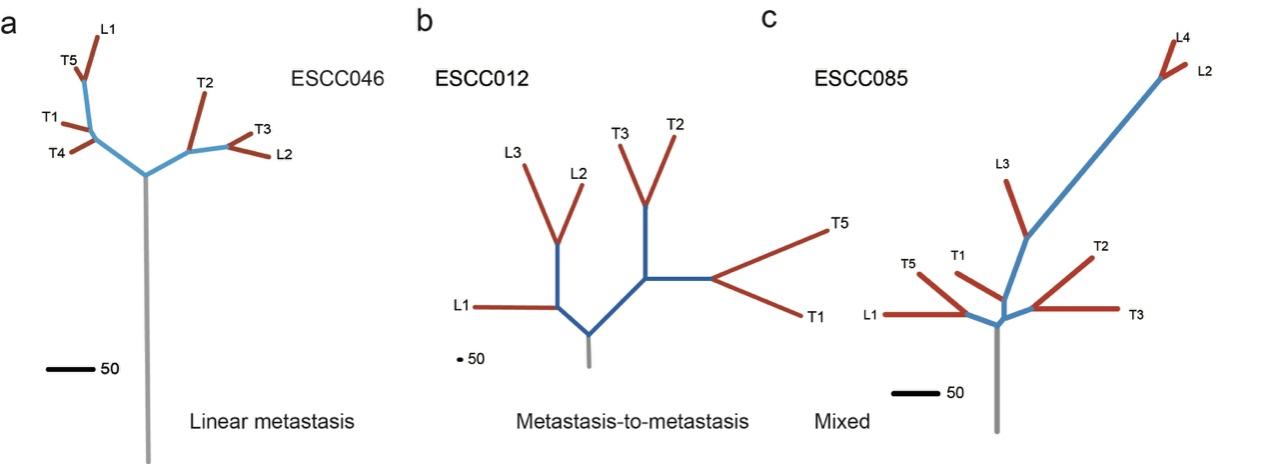


**Supplementary Figure 8.** Evolutionary trees of representative patients with distinct metastatic trajectories, including linear metastasis, metastasis-to-metastasis, and mixed metastasis. Gray represents the trunk mutations, blue represents the branch mutations, and red represents the private mutations in the evolutionary tree.


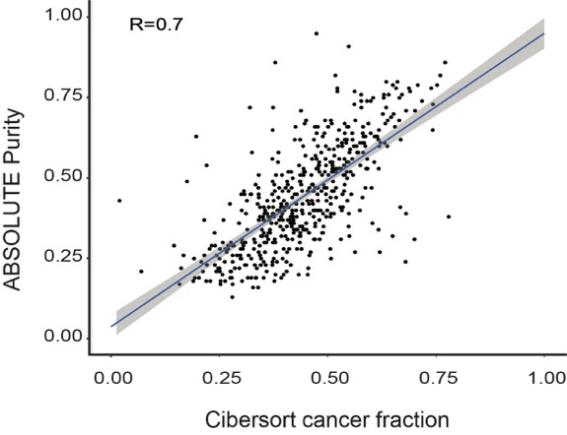


**Supplementary Figure 9.** Cancer cell fraction predicted by Cibersort-Methyl. Scatter-plot of the correlation (Spearman) between ABSOLUTE purity and Cibersort cancer fraction.


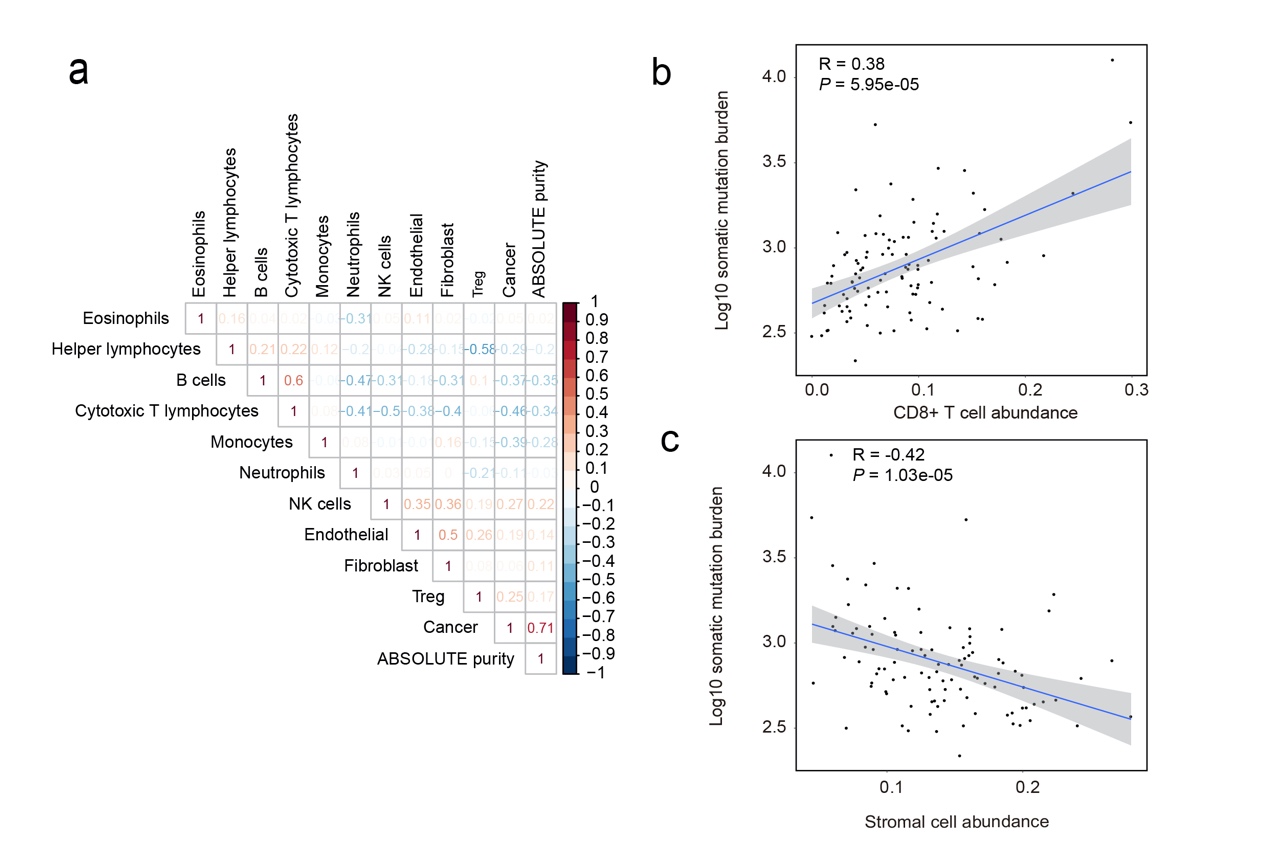


**Supplementary Figure 10. Cell components correlation in immune microenvironment. (a)** Correlation heatmap between different cell types and cancer. **(b)** Scatter-plot of the correlation (Spearman) between CD8+ T cell and somatic mutation burden. **(c)** Scatter-plot of the correlation (Spearman) between stromal cell and somatic mutation burden. Spearman’s correlation and P value are reported. The line indicates the linear regression and the gray shading indicates the 95% CI of the regression.


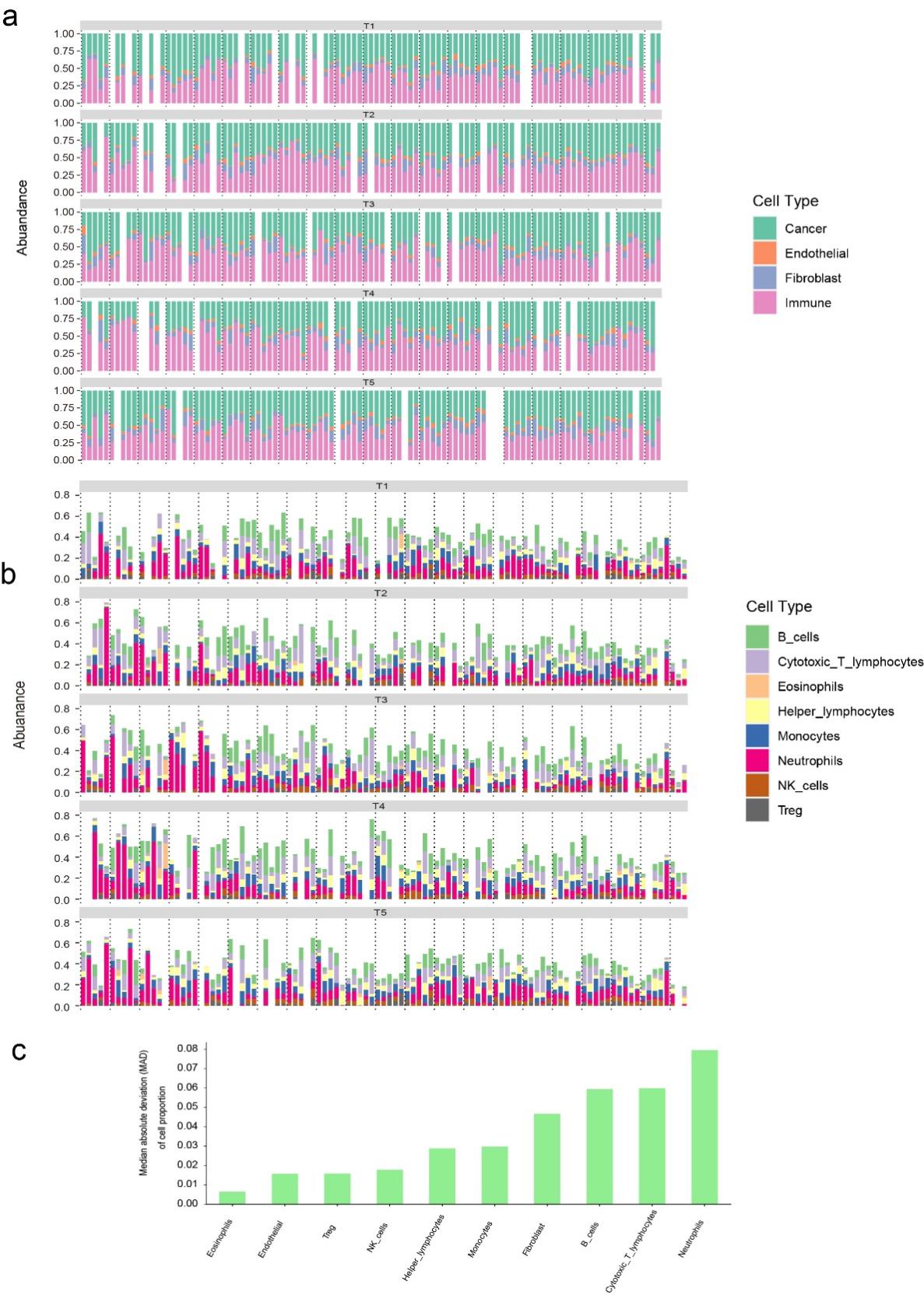


**Supplementary Figure 11. The proportion of cells in different regions of ESCC. (a)** Proportion of infiltrating cells in ESCCs. **(b)** Proportion of immune cell compositions in ESCCs. **(c)** The median absolute deviation (MAD) of cell proportion for each cell type.


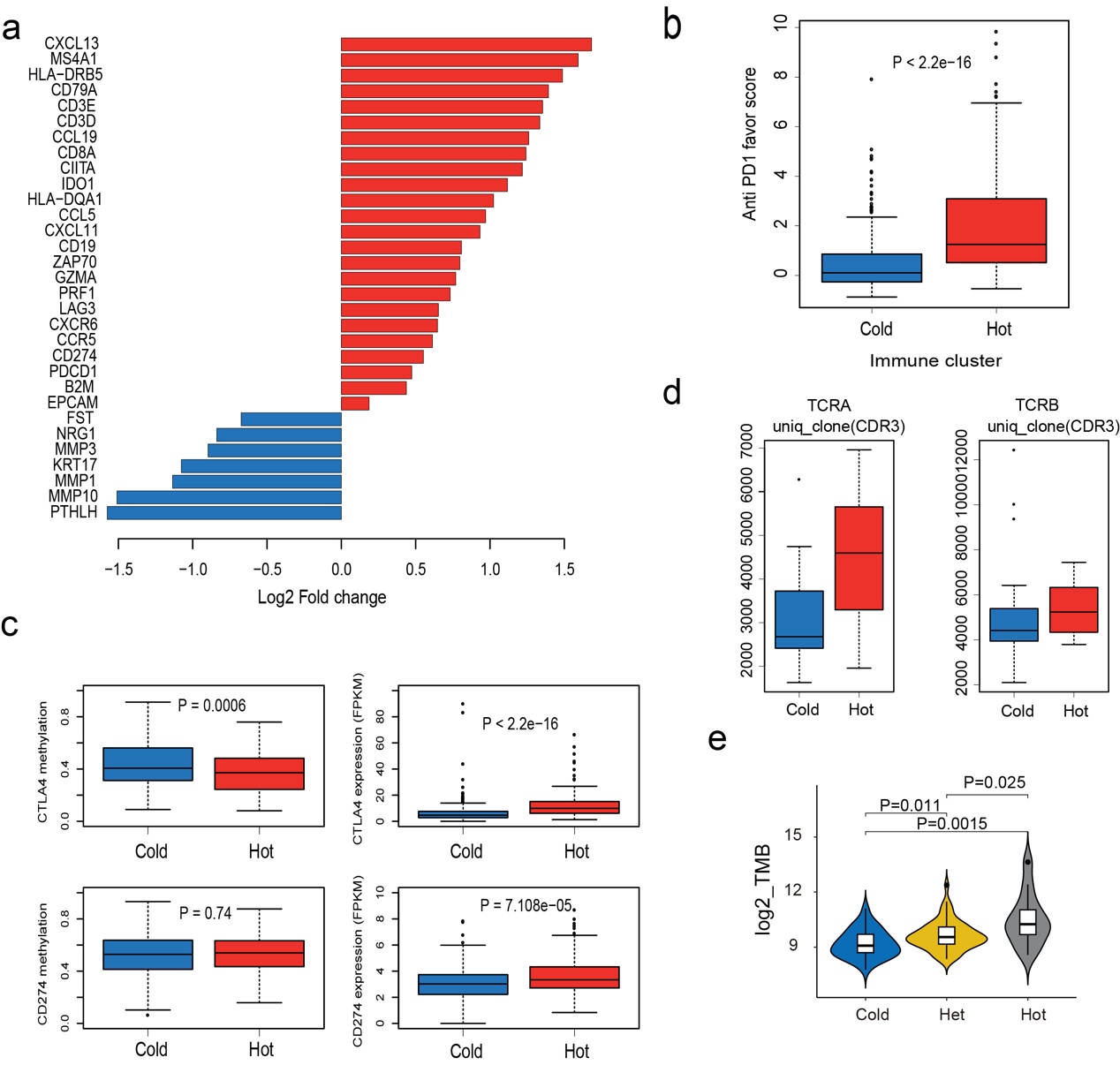


**Supplementary Figure 12. The difference between cold tumors and hot tumors. (a)** Differentially expressed genes between hot and cold tumors. **(b)** Box-plot showed anti-PD1 favor score between hot and cold tumors. **(c)** Box plots showed methylation and expression of *CD274* and *CTLA4* between hot and cold tumors. **(d)** Box plots showed TCRA and TCRB unique clones between hot and cold tumors. Box plot data are presented as the median ± standard deviation. The two-group test is based on the Wilcoxon test. **(e)** Violin plot of tumor mutation burden in different immune microenvironments.


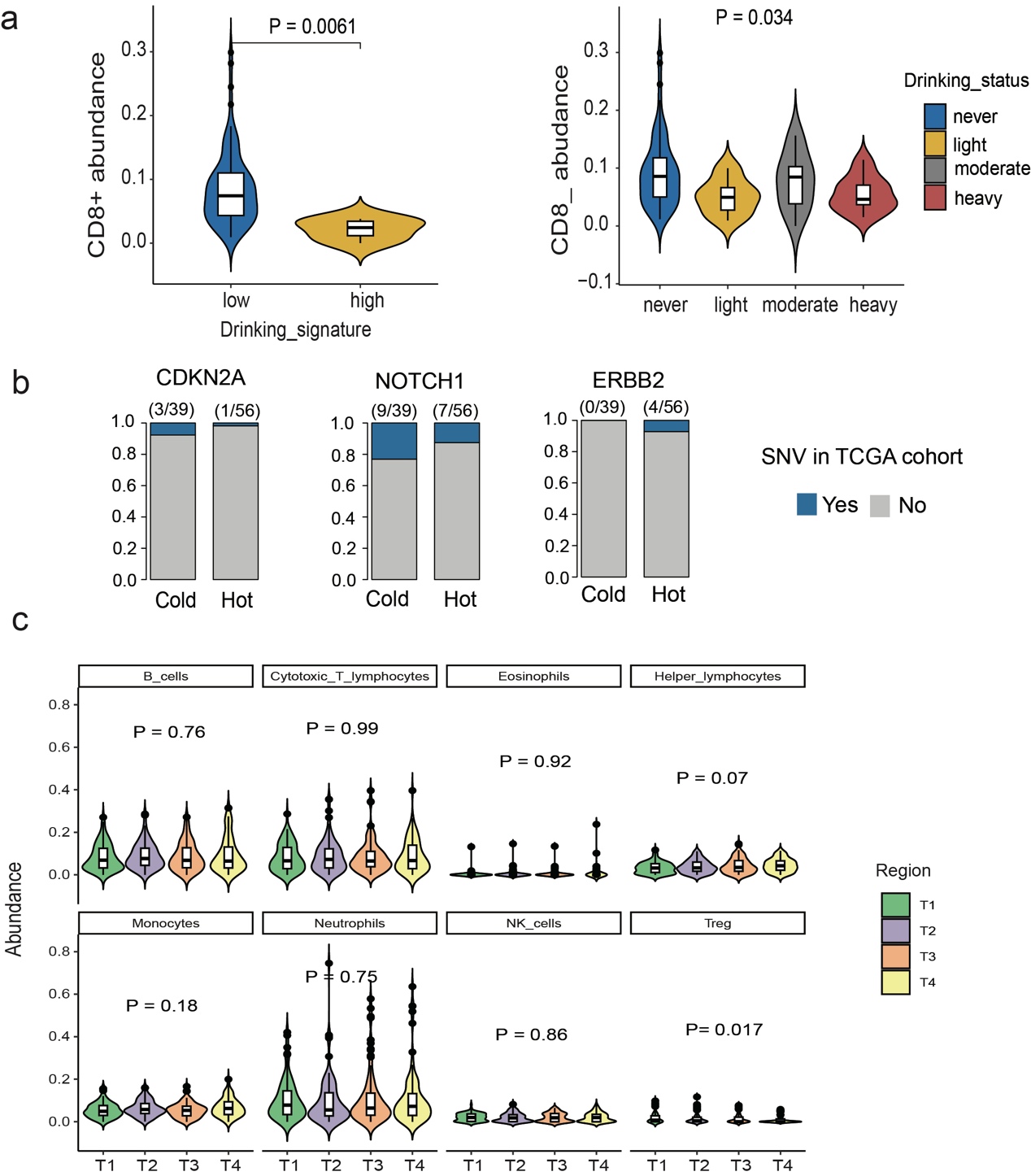


**Supplementary Figure 13. (a)** Violin-plot showed the correlation between CD8+ abundance and drinking. **(b)** The proportion of three gene mutations in hot and cold tumors (TCGA cohort). **(c)** Violin plots showed the immune infiltration in different tumor regions. The two-group test is based on the Wilcoxon test. The multi-group test is based on the Kruskal-Wallis test.


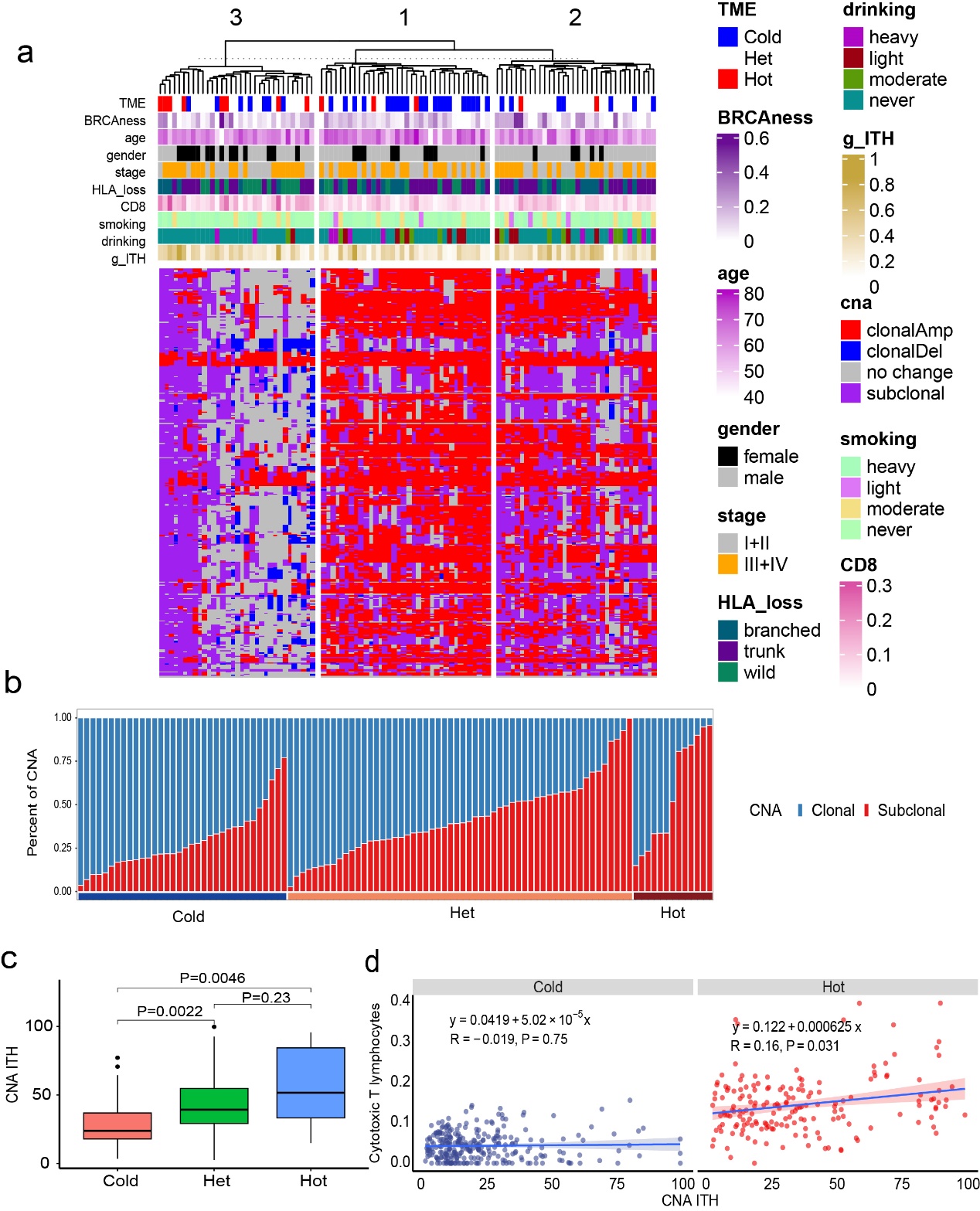


**Supplementary Figure 14. Somatic CNAs are associated with the immune microenvironment. (a)** The profile of clonal CNA and subclonal CNA for ESCCs. Immune type, CD8+, HLA status, gITH, and clinical characters were annotated in the panel. **(b)** Proportion of clonal CNA and subclonal CNA in ESCCs among three immune types. **(c)** Comparison of CNA ITH among three immune types. Statistical analysis is performed with a Two-sided Wilcox Rank-Sum Test. **(d)** The association between CNA ITH and the abundance of CD8+ cells at sample level in cold and hot tumor regions, respectively.


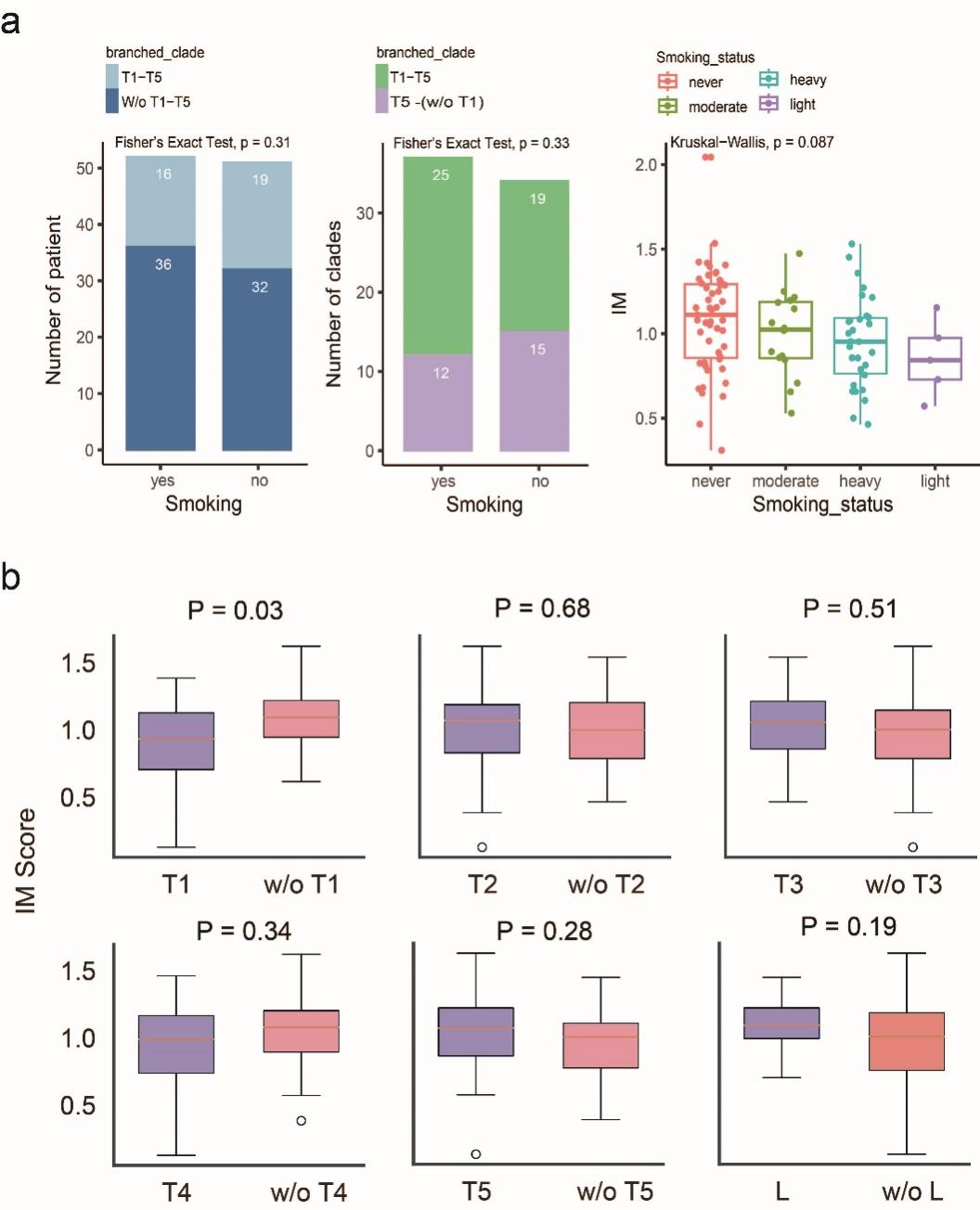


**Supplementary Figure 15. The association between IM score and smoking or geographical regions. (a)** The number of patients harbor branched clades involving T1 and T5 (left). The number of branched clades involving T5 and T1/other regions (w/o: without, middle). The IM score is across four smoking groups (right). **(b)** Box plots showed IM scores of branched clades in different ESCC regions. ‘w/o T1’ means the branched clads are not involved in the sub-region T1. On the boxplots, the horizontal line indicates the median, the box indicates the first to third quartile and the whiskers indicate 1.5 × the interquartile range. The two-group test is based on the Wilcoxon test.


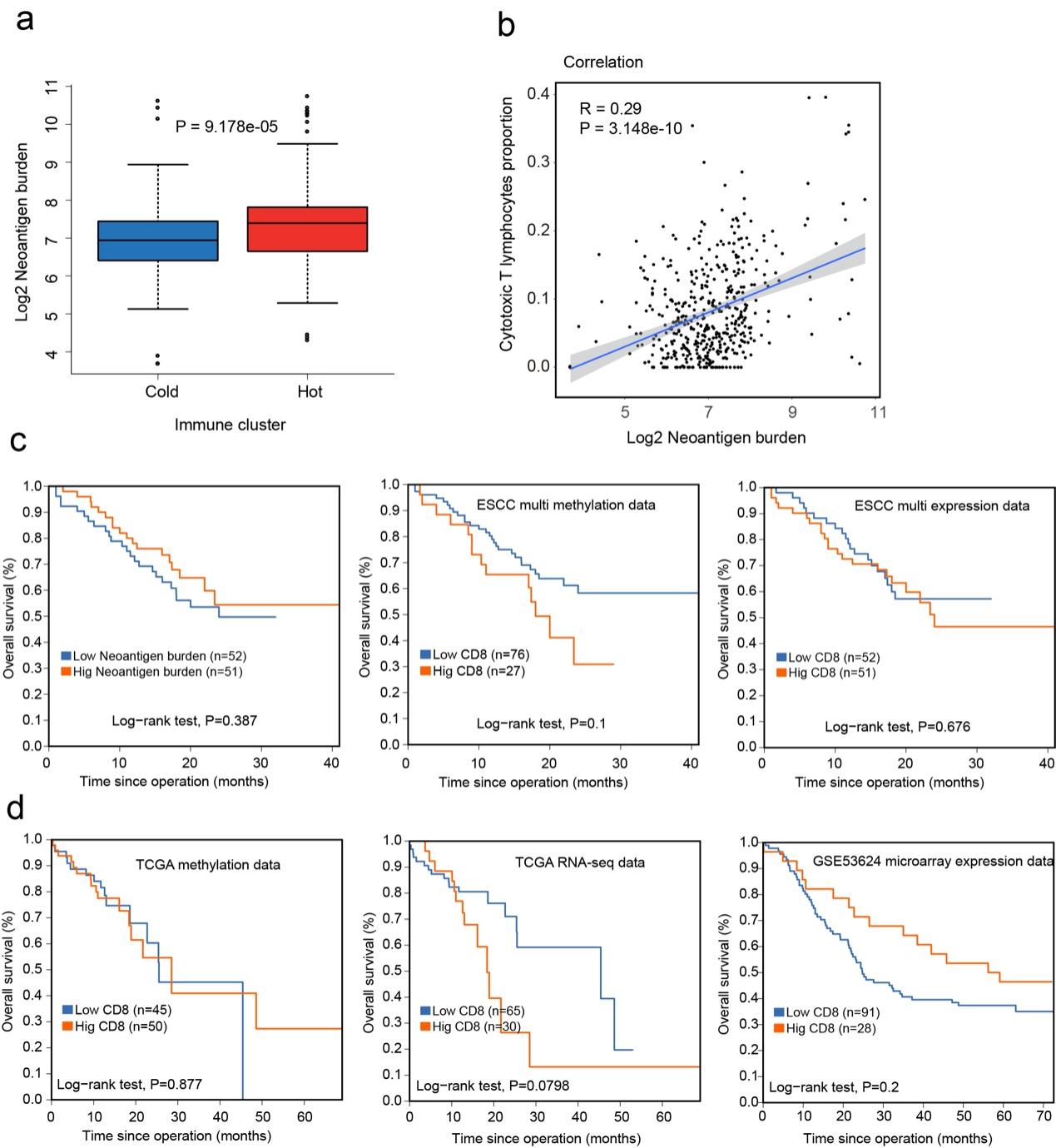


**Supplementary Figure 16. (a)** Box-plot showed the neoantigen burden between hot and cold tumors. The two-group test is based on the Wilcoxon test. **(b)** Scatter-plot of correlation between neoantigen burden and CD8+ cell composition. Spearman’s correlation and P value are reported. The line indicates the linear regression and the gray shading indicates the 95% CI of the regression. **(c)** The association of neoantigen burden and the abundance of CD8+ cells with overall survival in our data. Statistical analysis is performed with a Log-rank test. **(d)** The association of the abundance of CD8+ cells with overall survival in the validation cohort. Statistical analysis is performed with a Log-rank test.


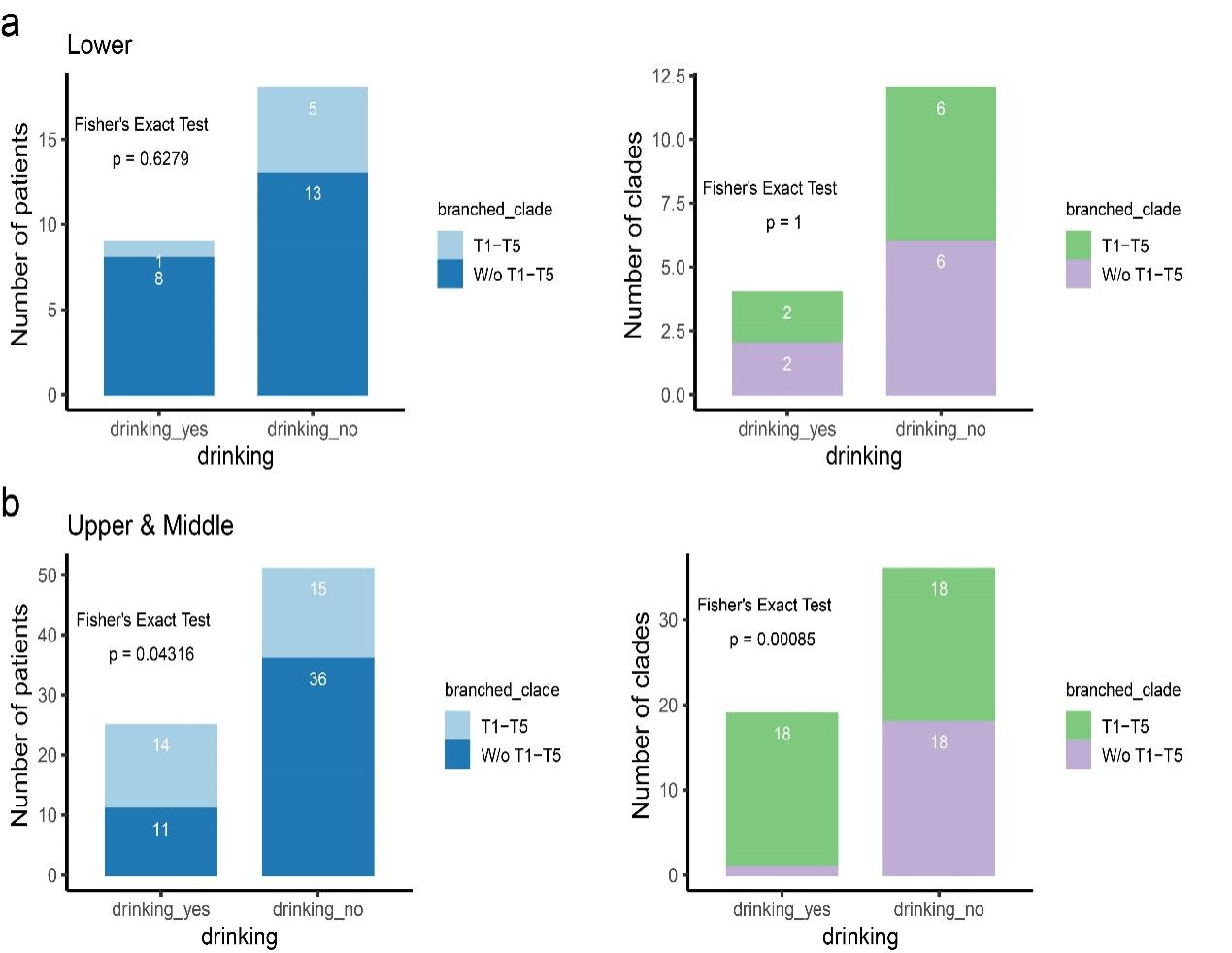


**Supplementary Figure 17**. **The spatial evolution in ESCCs of upper/middle and lower esophagus.** For the ESCC of lower **(a)** and upper/middle **(b)**, the number of patients having T1-T5 branched clades between drinkers and non-drinkers (left panel); The number of T1-T5 branched clades between drinkers and non-drinkers (right panel).


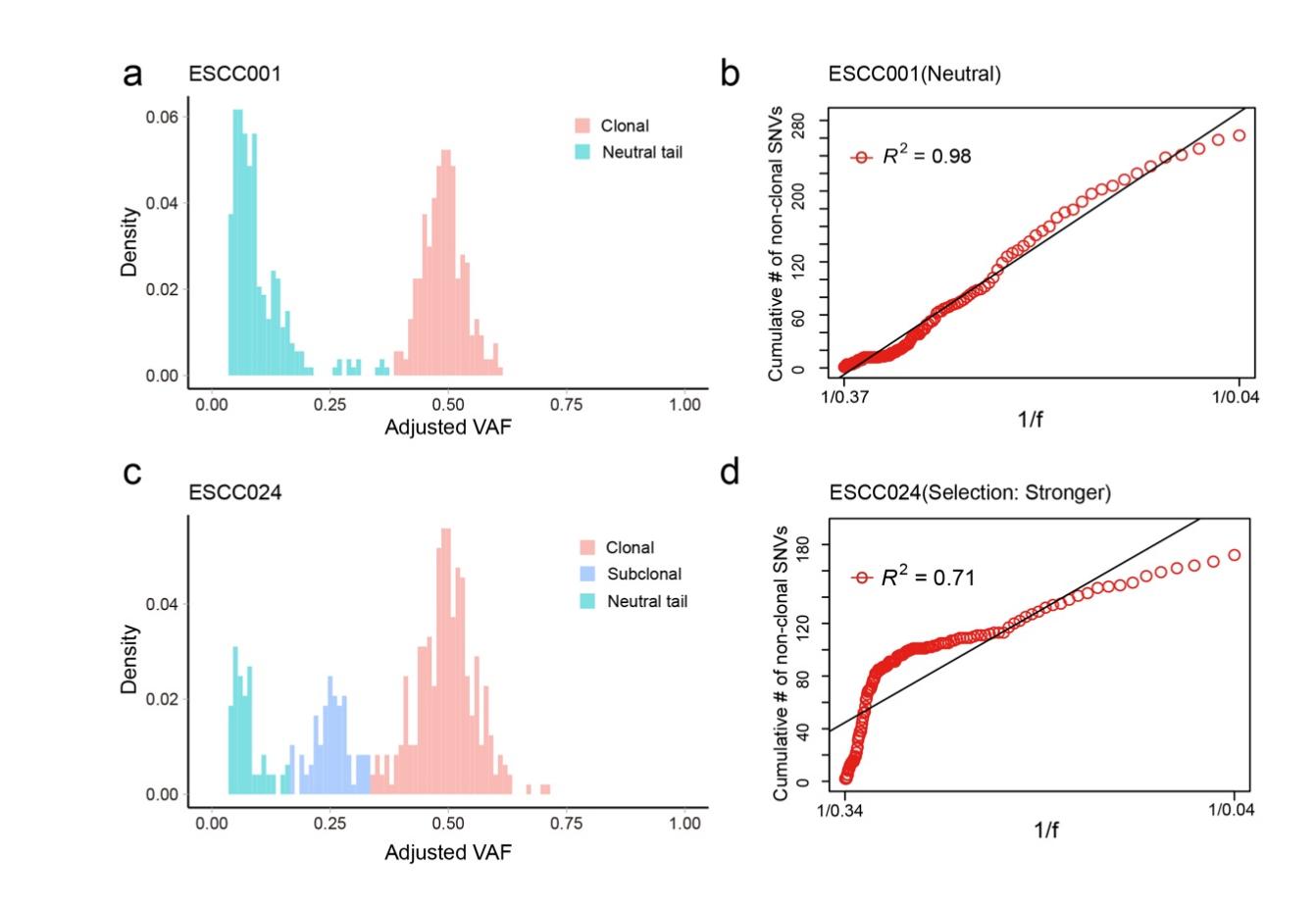


**Supplementary Figure 18.** **Examples of site frequency spectrum patterns and linear fitness for ESCCs with neutral and selection evolution.** **(a, c)** Analysis after MOBSTER identified one clonal cluster and a neutral tail of subclonal mutations in patient ESCC001 **(a)** in the VAF distribution, but one extra subclone private to patient ESCC024 **(c)** due to selection. **(b, d)** Correlations between inverse allele fraction (1/f ) and cumulative number of SNVs in patient ESCC001 **(b)** and ESCC024 **(d)**, respectively. The R-square represents the goodness of fit for the neutral evolution model (cutoff = 0.98).


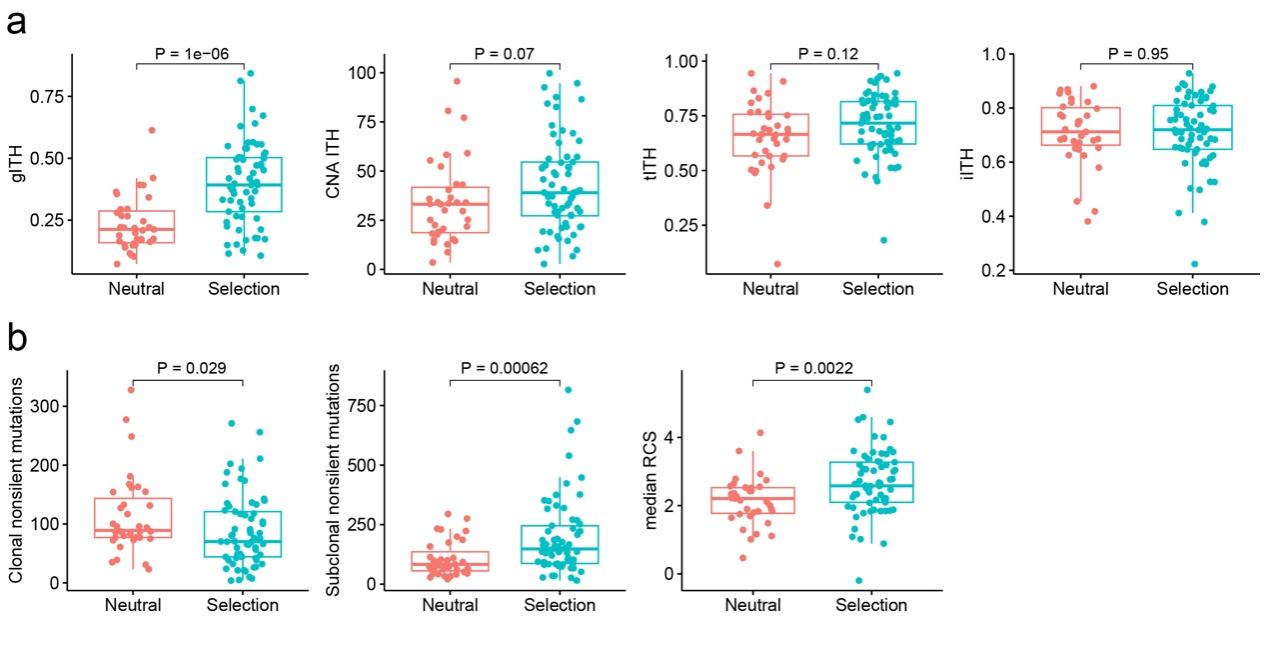


**Supplementary Figure 19. The genetic differences between neutral tumors and selective tumors.** **(a)** Boxplot showed gITH, CNA ITH, tITH, and iTH between neutral tumors and selective tumors. **(b)** Boxplot showed clonal/sub-clonal non-silent mutations, median regional clonality score (RCS) between tumors and selective tumors. Statistical analysis is performed with the Two-side Wilcox Rank-Sum Test.


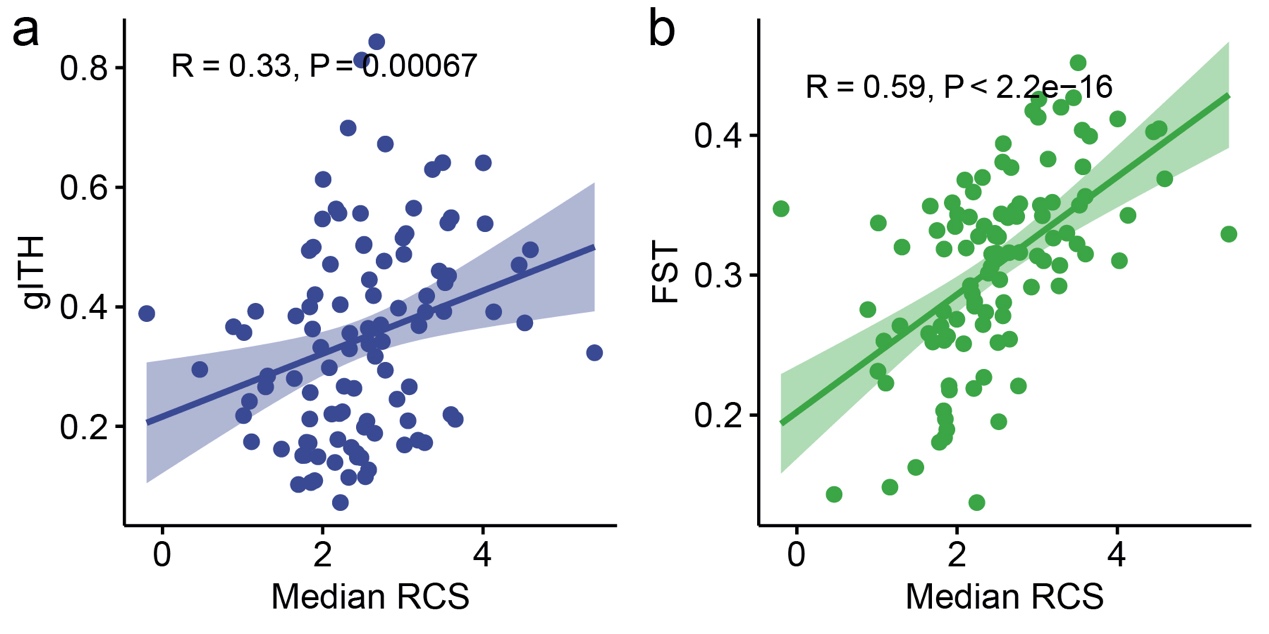


**Supplementary Figure 20. The selection pressure was related to intra-tumor heterogeneity. (a, b)** The scatter plots showed the correlation between RCS and gITH **(a)**, as well as between RCS and FST in ESCCs **(b)**, respectively. Spearman’s correlation and P value are reported. The line indicates the linear regression and the shading indicates the 95% CI of the regression.


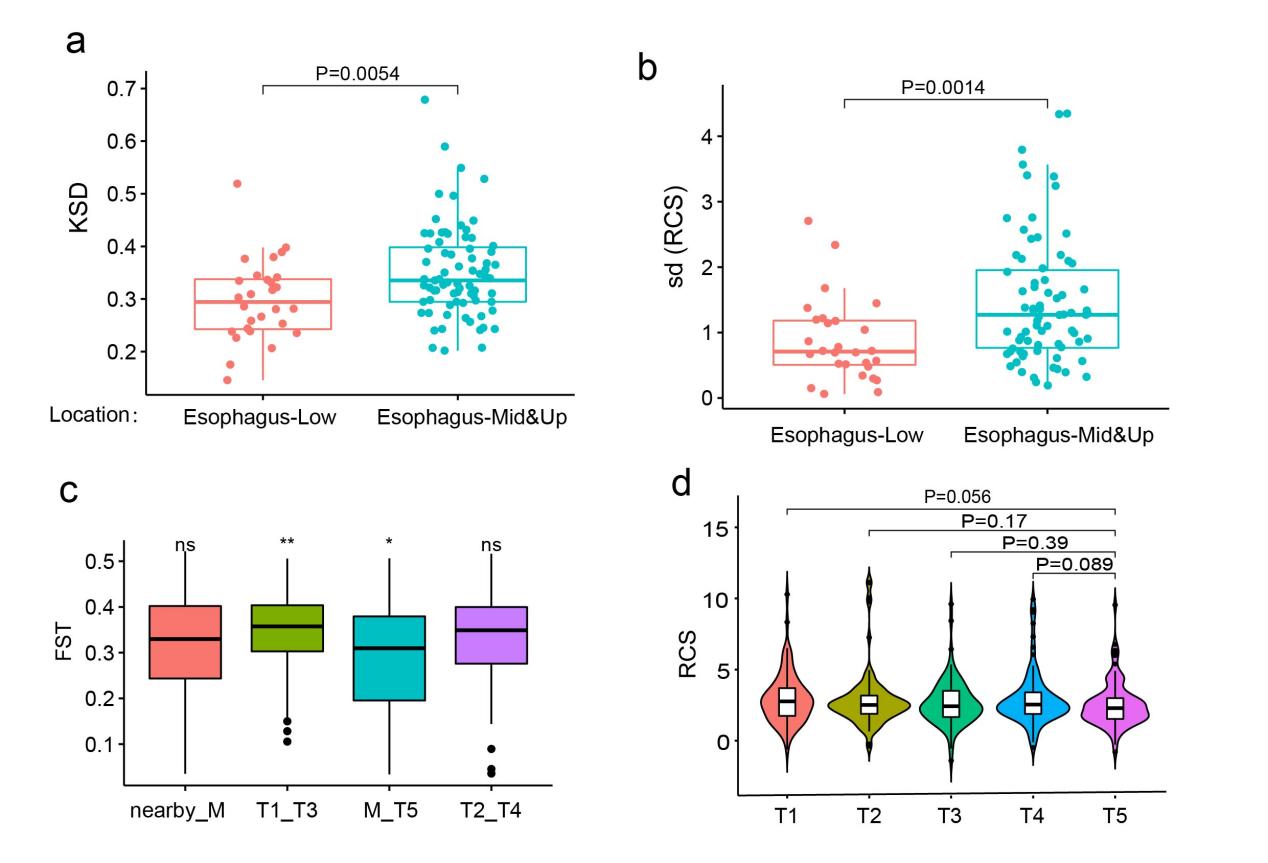


**Supplementary Figure 21. The spatial genetic heterogeneity between tumors and within tumors.** **(a)** Boxplot of KSD between lower and middle/upper tumors. **(b)** Boxplot of the standard derivation of RCS between lower and middle/upper tumor regions. **(c)** Comparisons of the FST among marginal regions. ‘M’ and ‘nearby_M’ represent the margin region in the primary tumor and adjacent margin region pairs, respectively. **(d)** Boxplot of RCS between tumor center (T5) and other different tumor regions. Statistical analysis is performed with the Two-side Wilcox Rank-Sum Test.


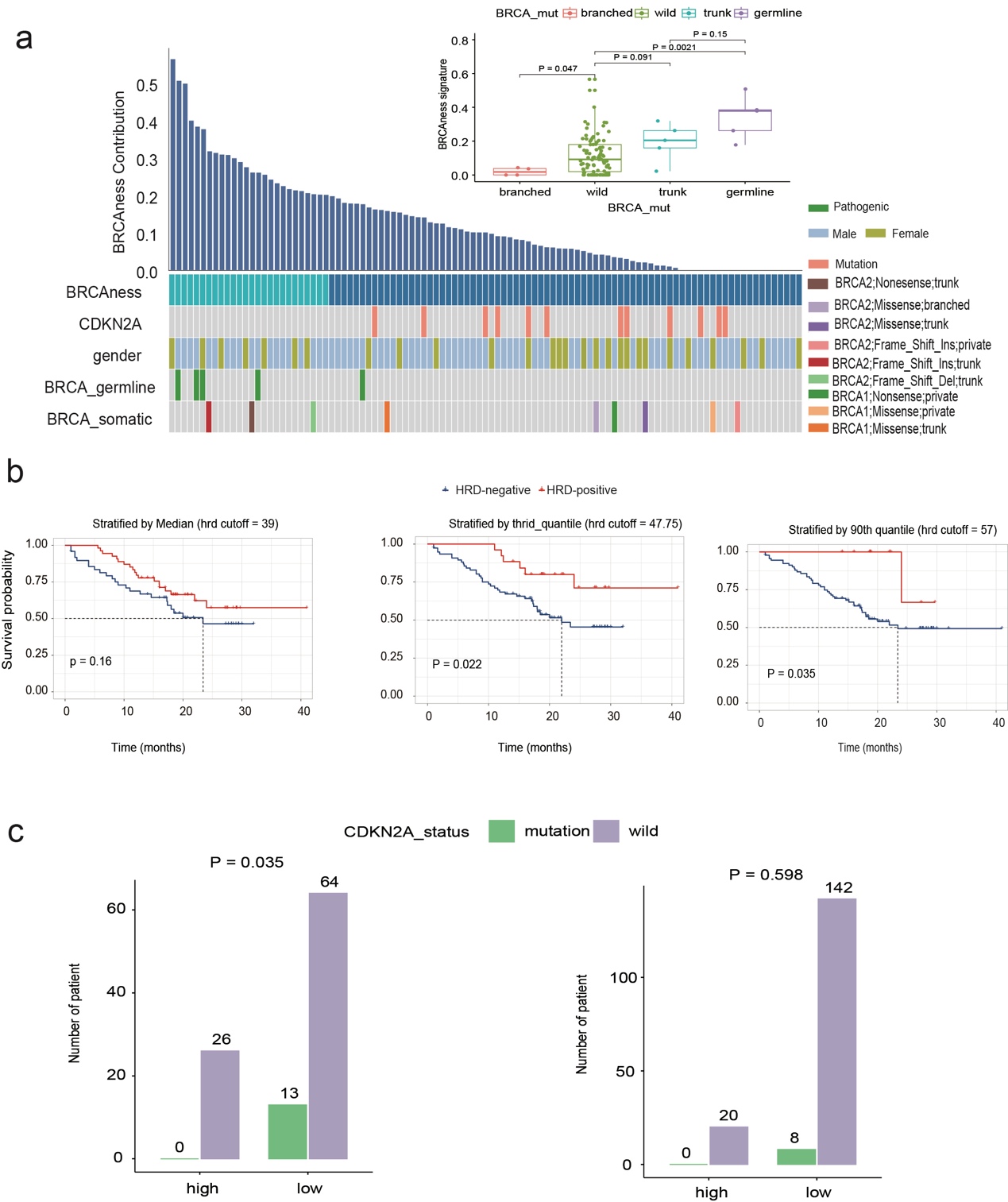


**Supplementary Figure 22. The association between BRCAness with genetic or clinical metrics.** **(a)** BRCAness signatures and *BRCA1/2* germline/somatic variants in 103 patients. P-values between patients with/without *BRCA1/2* are shown in the top boxplot. **(b)** The impact of HRD of different cutoffs (median, 75%, 90% quantile) on overall survival. Statistical analysis is performed with a Log-rank test. **(c)** The *CDKN2A* mutations were depleted in BRCAness patients in the main study cohort (left) and validation cohort (right). The two-group test is based on the Wilcoxon test.
