# Supplementary material for: Immune-tumor interaction dictates spatially directed evolution of esophageal squamous cell carcinoma
Source: Natl Sci Rev. 2024 Apr 23;11(5):nwae150. doi: 10.1093/nsr/nwae150 (PMC11129594; doi:10.1093/nsr/nwae150)
Supplement: nwae150_Supplemental_Files [file nwae150_supplemental_files.zip › Supplementary Notes.docx]

**Clinical samples**

Samples used in this study were obtained from 103 patients recruited from Shanxi province, China (**Fig. 1a**). All patients signed their informed consent, and all samples were obtained before treatment according to the guidelines of the local ethical committees. All ESCC patients were staged according to the Cancer Staging Standards of the American Joint Committee on Cancer. Primary tumor samples and matched adjacent histologically normal tissues were obtained and frozen into liquid nitrogen within 30 minutes post-surgery. The length, width, and depth of tissue samples were measured immediately after dissection in operation, and then five spatially isolated tumor specimens were obtained per patient, and each peripheral tumor sample was 0.5cm away from the central tumor sample. The tumor specimens were extracted from the tumor mass's bottom (T3), upper (T1), center (T5), left (T2), and right (T4). Meanwhile, we collected metastatic lymph nodes (1–3 samples per patient, from 24 patients). All samples were stocked in a −80 °C freezer until DNA extraction. After quality control of sequencing data, we removed those samples with low-quality data and performed analyses using the samples with good-quality data, which included 103 normal esophageal tissues and 507 regional tumors from 103 patients, and 46 metastatic lymph nodes samples (**Supplementary Data 2**). Detailed clinical characteristics of 103 ESCC individuals are provided in **Supplementary Data 1**. All research subjects provided written informed consent to participate in this study. The ethical committees of Shanxi Medical University approved the study.

**Whole-exome sequencing**

Genomic DNAs were extracted from regional tumors, and metastatic lymph nodes, and matched adjacent normal tissues. WES libraries were constructed as follows: a total amount of 0.6 μg genomic DNA per sample was used as input material for the DNA sample preparation. Sequencing libraries were generated using the Agilent SureSelect Human All Exon V6 kit (Agilent Technologies, CA, USA) following the manufacturer’s recommendations, and index codes were added to each sample. Briefly, fragmentation was carried out by the hydrodynamic shearing system (Covaris, Massachusetts, USA) to generate 180–280 bp fragments. DNA fragments with ligated adapter molecules were selectively enriched by PCR, and then exons of genes were captured with a biotin-labeled probe. Captured libraries were enriched by PCR to add index tags to prepare for sequencing. Products were purified using the AMPure XP system (Beckman Coulter, Beverly, USA) and quantified using the Agilent high-sensitivity DNA assay on the Agilent Bioanalyzer 2100 system. Then sequencing was performed on an Illumina HiSeq X-Ten sequencer with 150-bp paired-end reads.

**RNA-sequencing**

Total RNA was extracted from frozen samples using Trizol reagent (Life Technologies, Carlsbad, CA, USA) and DNA was digested with DNase I according to the manufacturer's instructions. The quantity and quality of RNA were evaluated by Nanodrop Spectrophotometer (Thermo Scientific, USA). RNA integrity was measured by 1% gel electrophoresis. mRNA was enriched with oligonucleotides (DT) and cleaved into fragments to prepare the cDNA library. The quality of cDNA library was checked by Agilent 2100 biological analyzer and ABI Step One Plus Real-Time PCR System, and then sequenced on Illumina Hiseq X Ten.

**Somatic mutation calling**

We applied the WES results of paired tumor and normal samples to call the somatic mutations. Raw reads of whole-exome sequencing were mapped to human reference (hg19) using BWA-mem^1^ with the default parameters. Picard ([www.broadinstitute.github.io/picard](http://www.broadinstitute.github.io/picard)) was used to mark duplicated reads. Somatic SNVs were called using mutect^2^ with default parameters. The supporting reads for putative SNVs were at least 4 in tumor BAM and one or less in normal BAM. This strategy helped exclude germline variants. Somatic Indels were called by using Mutect2 in GATK^3^, version 4.0.5.1, with supported reads >=4 and VAF >=0.1. In addition, we realized somatic mutations were probably missing in some regions owing to low purity. We also searched for supporting reads for mutations at the patient level in all the regions of the same patient using bam-readcount^4^. The mutations with VAF > 0.01 were regarded as positive ones and kept for follow-up analyses. The mutations were annotated with Oncotator^5^.

**Somatic copy number alteration calling**

Somatic copy-number alterations (CNAs) were detected using TitanCNA^6^ by comparing the normalized tumor and normal data, as previously described^7^. Only autosomes were used in copy number analysis. The sample purity values derived from ABSOLUTE^8^ were used as input, considering that called copy number alterations in this process were influenced by sample purity. TitanCNA was run with different numbers of subclones (n=1-3), and the optimal fitted output for each tumor was chosen by manual review. In principle, results from all samples of the same tumor were highly consistent. Overall tumor ploidy and purity for each sample were also re-calculated from the TitanCNA results (**Supplementary Data 8**), which are strongly associated with values inferred by ABSOLUTE.

We further classified somatic copy-number alterations, which were measured as a percentage of the genome affected by such alterations, as clonal (present in all regions) or subclonal (present in part of regions) as previous study^9^. In other words, any segment of gain or loss in the genome that overlapped across all regions was defined as clonal, and all other segments of copy number aberrations as subclonal, as shown in **Supplementary Figure 14a**. To measure the intra-tumor heterogeneity of CNAs (CNA ITH) for each tumor, we determined the proportion of subclonal CNAs in each region (**Supplementary Data 7**), then calculated the global proportion of subclonal CNAs within tumors (**Supplementary Data 3b**).

**Cancer cell fraction estimation**

In general, the cancer cell fraction (CCF) of each somatic SNV (SSNV) in autosomes was estimated by adjusting its observed VAF ($f$) based on local allele-specific copy numbers ($N_{T}$) and overall tumor purity ($\rho$). However, the number of mutant copies (also known as mutation multiplicity) present in cancer cells was not always one. Therefore, it was necessary to further adjust CCF for mutation by its multiplicity^10^. Thus, CCF for each SSNV at sample-level is calculated as:

$CCF=\frac{f}{m\rho}\left( \rho N_{T}+2\left( 1-\rho\right) \right)$ Eq. (1)

Where $m$ indicates mutation multiplicity inferred as:

$m=\max\left( 1, round\left( \frac{f}{\rho}\left( \rho N_{T}+2\left( 1-\rho\right) \right) \right) \right)$ Eq. (2)

The merged CCF of each SSNV is computed by integrating CCF values from multiple regions of the same tumor when the multi-region sequencing (MRS) data is available:

$\bar{CCF}=\left\{ \begin{aligned} \frac{\sum_{i=1}^{k} {CCF}_{i}\times d_{i}}{\sum_{i=1}^{k} d_{i}} ,\bar{CCF}<1 \\ 1 ,\bar{CCF}\geq1 \end{aligned} \right.$ Eq. (3)

where $d_{i}$ and ${CCF}_{i}$ are the sequencing depth and cancer cell fraction estimation in region $i$, respectively. It is worth noting that the adjusted VAF value of each SSNV is half of its CCF value.

**The estimation of the genetic distance**

The subclonal SSNVs can directly reflect the dynamics of clonal expansion and intra-tumor heterogeneity (ITH) after tumor transformation. An SSNV in a primary tumor is defined as subclonal in the MRS data if all of the following criteria: 1) the adjusted VAF of at least one region should be larger than 0.04; 2) the adjusted weighted VAF for a whole tumor is lower than 0.25; 3) At least one region with the adjusted VAF < 0.1. An SSNV that does not meet one of the above criteria will be considered clonal. Except for the fraction of subclonal SSNVs, we measured the levels of ITH in DNA based on the subclonal SSNVs for pairwise comparisons of regions^11^. We applied FST and KSD to define the genetic divergence and distribution similarity between regions and calculated the average of FST and KSD for all combinations of sample pairs in whole tumors (Supplementary Data 6, 9).

**Mutational signatures**

To compute the underlying mutational signatures in each region or patient, we apply MutationalPatterns^12^ to the mutations of each region or patient. The proportion of thirty mutational signatures was quantified with the default parameters. Patient-level mutations are unique mutations from all regions. The annotation of each signature was based on COSMICV2.

**HRD score**

We employed the Sequenza^13^ to compute the allelic copy numbers. scarHRD^14^ was used to quantify the score of HRD score with the default parameters. The patient-level HRS score is the average of the HRD scores of all sub-regions from a patient.

**ITH quantification**

Immune ITH (iITH) and transcriptome ITH (tITH) were quantified by MetaITH^15^. The VAFs of somatic SNVs in each sample were converted into binary value. The pairwise distance of somatic SNVs between sub-regions are calculated. The gITH was the average of the pairwise distances from different sub-regions for each patient.

**Gene expression quantification**

Raw RNA-sequencing reads were subjected to SOAPnuke (v1.5.0; https://github.com/BGI-flexlab/SOAPnuke) to remove sequencing adapters, and low-quality reads. Clean reads were aligned with bowtie2, version2.3.4^16^, and gene values were quantitated with RSEM, version1.3.1^16^, with default parameters and count upper-quartile normalized and log2 transformed for analysis.

**Methylation analysis**

We applied the ChAMP package^17^ on the raw 850K IDAT array data to evaluate the methylation level of the tumor and matched normal samples. We processed data loading, quality control, sample exclusion, intra-sample probe normalization, and statistical analysis with default parameters. From the results, we got the beta value, which was the ratio of the intensity of the methylated probe intensity versus the intensity of the total probe, as the methylation level of each probe. The resulting 665413 probes were mapped to the human genome (GRCh38) using the cpg.annotate R package. We then identified the probes in a promoter region of the UCSC gene. Suppose there was one gene promoter associated with multiple probes. In that case, we choose the probe of which the methylation beta values are most negatively correlated with the gene expression profile to be the methylation level of the associated gene. These presentative probes were also used to distinguish hyper or hypo methylation at the sample level. Specifically, gene hypermethylation was defined as the mean beta value of tumor regions from one patient for the representative prob being 0.3 greater than the mean beta value in the normal sample. In addition, the mean beta value of the normal samples is smaller than 0.3. In contrast, gene hypomethylation was defined as the mean beta value of tumor regions from one patient for the representative prob being 0.3 smaller than the mean beta value in the normal sample. In addition, the mean beta value of the normal samples was greater than 0.6.

**Phylogenetic tree construction**

The VAFs of SSNVs of sub-regions from each patient were converted into the binary table that was utilized by Discrete Character Parsimony, implemented in PHYLogeny Inference Package (<http://evolution.genetics.washington.edu/phylip.html>) to produce the phylogenetic tree. We required that the trunk, branched and private clade consist of at least 10 coding mutations, which makes it practical to estimate the IM score.

**Tumor microenvironment deconvolution**

The benchmark by [Chakravarthy](https://www.nature.com/articles/s41467-018-05570-1#auth-Ankur-Chakravarthy-Aff1-Aff7)^18^ et, al revealed that methylation data is more accurate for deconvolution compared to transcription data. Together with the observation that the correlation between cancer purity predicted from DNA methylation data and the purity predicted from ABSOLUTE is consistent. Hence, we used the signature matrix of ESCC in the MethylCIBERSORT R package^18^ for deconvolution. The beta values of the signature probe for deconvolution-associated features were then generated. Moreover, the signature matrix and beta values profile were uploaded to the CIBERSORT website (https://cibersortx.stanford.edu/) to infer the composition of 11 cell types. We run CIBERSORT by using 100 permutations with default parameters. Result files were downloaded from the CIBERSORT website as tab-delimited text files and were imported into R for downstream analysis.

**Neoantigen prediction**

We inferred the HLA status for each sample by using HLA-VBSeqv2^19^. This method uses variational Bayesian inference to optimize the abundance of reads on HLA alleles and the read alignments of HLA allele sequences. MHC binding affinities were inferred as IC50 values for the patient HLA type and each peptide sequence. All mutant peptide sequences meet a standard cut-off: the IC50 of mutant peptide < 500nM and the IC50 of the wild peptide > 500nM were considered to be potential neoantigens. NetMHCpan4.0^20^ was used to predict Peptide-MHC class I binding affinity, while NetMHCIIpan-3.0^21^ was applied to identify peptides that bind to MHCII molecules.

**Immunoediting score**

We computed immunoediting scores based on the observed neoantigen and non-synonymous mutations and expected ones. We utilized a similar strategy to calculate the score with a modification of a previously published method^22^. The probability of a coding mutation becoming immunogenic is estimated based on all mutations and neoantigens of 103 patients, denoted as B. The likelihood of coding mutations becoming non-synonymous depends on the mutation context, denoted as M, computed based on 103 patients' mutations. Regarding the immunoediting score of the clone, the computation formula was followed.

$expected\_nony=\sum_{i} M\left( i \right)$ ; $expected\_neoantigen=N*B$ (N: number of coding mutations)

$$IM\_score= \frac{observed\_neoantigen/observed\_nonsy}{expected\_neoantigen/expected\_nonsy}$$

**Quantifying the regional clonality score within the tumor**

To investigate the spatial selective pressure over the tumor area, we defined a novel evaluation index - regional clonality score (RCS). RCS represents the quotient of the normalized number of major subclonal mutations within each section divided by the distance from the major subclone peak to the clonal peak, as shown in **Figure 5a**. We expressed the Log RCS as a function of the dynamics of subclonal expansion in region $i$ as follows:

${RCS}_{i}=log2\frac{{M_{i}}/{\sum_{j=1}^{k} M_{j}}}{d_{i}}$ Eq. (4)

Where $M_{i}$ and $M_{j}$ represent the subclonal mutations burden in region $i,$ and $j$ excluded the neutral tails identified by MOBSTER^23^; $d_{i}$ is the distance from the average of adjusted VAF of the major subclone to 0.5 (the default for the clonal peak is 0.5). Therefore, we can estimation the RCS value for each region (**Supplementary Data 7**). And the spatial expectation and variance of RCS across all regions within tumors were then calculated (**Supplementary Data 6**), which measure the degree of subclonal expansion and divergence among the whole tumor, respectively.

**The pooled site frequency spectrum (SFS) and neutrality**

To discriminate between neutral and non-neutral evolution, we applied MOBSTER^23^ to define clone clusters from the pooled SFS of somatic mutations, which could explicitly model co-existing neutral (Pareto Type-I distribution) and positive selection (Beta distribution) in whole cancer. The beta component parameter kBeta = 1 corresponds to neutral evolution. To ensure the reliability of the neutrality prediction, the percent of fitted neutral tails was at least 10%, and the mean VAF for clonal mutation was approximately 0.4. In addition, we also used the linear regression $1/f$ model^24^ to fit the cumulative number of only subclonal SSNVs ranged from 0.06 to 0.3. The tumors with the goodness-of-fit R^2^ >= 0.98 tend to have a good fit to the neutral expectation, consistent with the results from Mobster. For ESCC049, the subclone predicted by MOBSTER was more like a neutral tail, of which R^2^ = 0.98, so we argue it was more neutral. Finally, 37 neutral tumors are identified in our ESCC cohort (**Supplementary Data 6**).

**Screen for positive selection at the gene level**

To identify genes under significant positive selection, we first scanned for our ESCC cohort the presence of somatic mutations in esophageal-associated driver genes (defined on the basis of IntOGen) and focused on the top 50 driver genes. We then used the dNdScv tool by Martincorena et al^25^ to estimate their dN/dS ratio of non-synonymous to synonymous substitutions (pooled) for patients. Further, dN/dS values for the top 50 driver genes were extracted. In addition to global dN/dS estimates of the fitted models, dN/dS estimates of driver mutations were obtained with the ‘genesetdnds’ function of dndscv. The presence of possible positive selection would be supported by estimated values strictly greater than 1 (dN/dS > 1); neutral mutations should have dN/dS ≈ 1, and mutations under negative (or purifying) selection would show dN/dS < 1. Finally, Genes found as significant (q-value < 0.05) in ESCC are depicted in **Figure 1c**.

**Integrative timing**

For driver point mutations and recurrent copy number alterations, an ordering was first established for each tumor by PhylogicNDT. Then, the ordering results were aggregated across all tumors and the overall ranking of lesions was calculated by a sports statistics model as in previous studies^26^.

**Cell culture conditions**

The ESCC cell lines and the normal esophageal squamous cell lines used in this study were stored in our laboratory. All of the ESCC cell lines were cultured in HyClone™ RPMI-1640 medium (GE Healthcare Life Sciences, HyClone Laboratories, Logan, UT, USA) containing 10% fetal bovine serum (FBS; Gibco; Thermo Fisher Scientific, Inc, Waltham, MA, USA). The normal esophageal squamous cell line HET-1A was cultured in DMEM supplemented with 10% FBS. The normal esophageal squamous cell line NE3 was cultured in a 1:1 mixture of defined keratinocyte serum-free medium (dKSFM; Gibco; Thermo Fisher Scientific, Inc., Waltham, MA, USA) and EpiLife medium (Cascade Biologics, Inc., Portland, OR, USA). All of the cell lines were placed in an incubator containing 5% CO_2_ at 37˚C. The culture medium was replaced and the subculture was carried out according to the cell state and cell density respectively.

**Knockdown of PREX2 in ESCC Cell Lines**

We used the small interfering RNA (siRNA) for endogenous PREX2 knockdown. The sequence of PREX2-siRNAs was as follows: PREX2-si1: CCCAGTACTAGAGACTTGT and PREX2-si2: CCTTATACCACAGCACATA. According to the instruction, these siRNAs were transfected into the cells using riboFECT^TM^ CP-Reagent (RIBOBIO, Guangzhou, China). The knockdown efficiency was determined by RT-qPCR.

**RNA extraction and Real-time PCR**

The total RNA of ESCC cell lines was extracted using RNAiso plus (Takara, Dalian, China) according to the protocol. Complementary DNA (cDNA) was synthesized using a PrimeScript® RT reagent Kit with gDNA Eraser (Takara, Dalian, China) and TB Green® Premix Ex Taq® II kit (Takara, Dalian, China) was used for RT-qPCR. The primer sequences of GAPDH and PREX2 used in this study were as follows Table. Normalize GAPDH expression to determine the relative expression of PREX2 and calculated by using the 2-ΔΔCt formula.

The primer sequences of GAPDH and PREX2 used in this study

| **Gene name** | **Forward** | **Reverse** |
| --- | --- | --- |
| PREX2 | AAGACCGAGCGGGACTATGT | TGTTGAGCATTAGGTTCGGGG |
| GAPDH | GAAGGTGAAGGTCGGAGTC | GAAGATGGTGATGGGATTTC |

**Cell migration and invasion assay**

Migration and Invasion assays were performed by using transwell plates (8 µm, Corning, Inc.). 50,000 cells/well were seeded into the upper compartment of the transwell plates and cultured serum-free culture. The lower compartment of the plates was filled with culture with 10% FBS. For invasion assays, the upper cavity was coated with BD Matrigel Matrix which was diluted at 1:6 in advance with the serum-free medium. For migration assays, the above steps were not necessary. After 24h or 48h cultured, the upper cells were discarded. The cells passed through the membrane were fixed with 4% formaldehyde, stained with 0.1% crystalline amethyst, and counted using a microscope.

**CCK8 assay**

5,000 cells/well were seeded into a 48-well plate, with a final volume of 200 μL/well. After 24h, 48h, 72h, and 96h cultured, 20 μL/well CCK8 solution was added to each well and then the 48-well plate was cultured in the incubator at 37°C for 30 minutes. The absorbance of each well was determined at 450nm using a spectrometer.

**Colony formation assay**

1,000 or 1500 cells/well were seeded into a 6-well plate at 37°C with 5% CO2 for 10-15 days. Replace the culture medium according to the cell state and continue to culture the cells until the number of cells in a single clone is more than 50. Clean the cells surface with PBS and fix the cells with 4% paraformaldehyde for 30 minutes, then dye the cells with 0.25% crystal violet solution for at least 30 minutes and count under the microscope.

**Statistics**

The analyses consisting of statistical significance and plotting are primarily based on R (version 4.0) programming. The two-group test is based on the Wilcoxon test and p < 0.05 is defined as significance. The correlation between the two groups is based on Spearman in default. Survival analyses is based on the R package ‘survminer’ and ‘survival’.

1. Xie, C. *et al.* Fast and accurate HLA typing from short-read next-generation sequence data with xHLA. *Proc Natl Acad Sci U S A* **114**, 8059-8064 (2017).

2. Cibulskis, K. *et al.* Sensitive detection of somatic point mutations in impure and heterogeneous cancer samples. *Nat Biotechnol* **31**, 213-9 (2013).

3. McKenna, A. *et al.* The Genome Analysis Toolkit: a MapReduce framework for analyzing next-generation DNA sequencing data. *Genome Res* **20**, 1297-303 (2010).

4. Khanna, A. *et al.* Bam-readcount -- rapid generation of basepair-resolution sequence metrics. *ArXiv* (2021).

5. Ramos, A.H. *et al.* Oncotator: cancer variant annotation tool. *Hum Mutat* **36**, E2423-9 (2015).

6. Ha, G. *et al.* TITAN: inference of copy number architectures in clonal cell populations from tumor whole-genome sequence data. *Genome Res* **24**, 1881-93 (2014).

7. Hu, Z. *et al.* Quantitative evidence for early metastatic seeding in colorectal cancer. *Nat Genet* **51**, 1113-1122 (2019).

8. Carter, S.L. *et al.* Absolute quantification of somatic DNA alterations in human cancer. *Nat Biotechnol* **30**, 413-21 (2012).

9. Jamal-Hanjani, M. *et al.* Tracking the Evolution of Non-Small-Cell Lung Cancer. *N Engl J Med* **376**, 2109-2121 (2017).

10. Tarabichi, M. *et al.* A pan-cancer landscape of somatic mutations in non-unique regions of the human genome. *Nat Biotechnol* **39**, 1589-1596 (2021).

11. Sun, R. *et al.* Between-region genetic divergence reflects the mode and tempo of tumor evolution. *Nat Genet* **49**, 1015-1024 (2017).

12. Manders, F. *et al.* MutationalPatterns: the one stop shop for the analysis of mutational processes. *BMC Genomics* **23**, 134 (2022).

13. Favero, F. *et al.* Sequenza: allele-specific copy number and mutation profiles from tumor sequencing data. *Ann Oncol* **26**, 64-70 (2015).

14. Sztupinszki, Z. *et al.* Migrating the SNP array-based homologous recombination deficiency measures to next generation sequencing data of breast cancer. *NPJ Breast Cancer* **4**, 16 (2018).

15. Sharma, A. *et al.* Non-Genetic Intra-Tumor Heterogeneity Is a Major Predictor of Phenotypic Heterogeneity and Ongoing Evolutionary Dynamics in Lung Tumors. *Cell Rep* **29**, 2164-2174 e5 (2019).

16. Langmead, B. & Salzberg, S.L. Fast gapped-read alignment with Bowtie 2. *Nat Methods* **9**, 357-9 (2012).

17. Morris, T.J. *et al.* ChAMP: 450k Chip Analysis Methylation Pipeline. *Bioinformatics* **30**, 428-30 (2014).

18. Chakravarthy, A. *et al.* Pan-cancer deconvolution of tumour composition using DNA methylation. *Nat Commun* **9**, 3220 (2018).

19. Nariai, N. *et al.* HLA-VBSeq: accurate HLA typing at full resolution from whole-genome sequencing data. *BMC Genomics* **16 Suppl 2**, S7 (2015).

20. Jurtz, V. *et al.* NetMHCpan-4.0: Improved Peptide-MHC Class I Interaction Predictions Integrating Eluted Ligand and Peptide Binding Affinity Data. *J Immunol* **199**, 3360-3368 (2017).

21. Nielsen, M. & Andreatta, M. NetMHCpan-3.0; improved prediction of binding to MHC class I molecules integrating information from multiple receptor and peptide length datasets. *Genome Med* **8**, 33 (2016).

22. Angelova, M. *et al.* Evolution of Metastases in Space and Time under Immune Selection. *Cell* **175**, 751-765 e16 (2018).

23. Caravagna, G. *et al.* Subclonal reconstruction of tumors by using machine learning and population genetics. *Nature Genetics* **52**, 898-907 (2020).

24. Williams, M.J., Werner, B., Barnes, C.P., Graham, T.A. & Sottoriva, A. Identification of neutral tumor evolution across cancer types. *Nat Genet* **48**, 238-244 (2016).

25. Martincorena, I. *et al.* Universal Patterns of Selection in Cancer and Somatic Tissues. *Cell* **171**, 1029-1041 e21 (2017).

26. Gerstung, M. *et al.* The evolutionary history of 2,658 cancers. *Nature* **578**, 122-128 (2020).
